# Author Correction: Opportunistic detection of type 2 diabetes using deep learning from frontal chest radiographs

**DOI:** 10.1038/s41467-024-49184-2

**Published:** 2024-06-06

**Authors:** Ayis Pyrros, Stephen M. Borstelmann, Ramana Mantravadi, Zachary Zaiman, Kaesha Thomas, Brandon Price, Eugene Greenstein, Nasir Siddiqui, Melinda Willis, Ihar Shulhan, John Hines-Shah, Jeanne M. Horowitz, Paul Nikolaidis, Matthew P. Lungren, Jorge Mario Rodríguez-Fernández, Judy Wawira Gichoya, Sanmi Koyejo, Adam E Flanders, Nishith Khandwala, Amit Gupta, John W. Garrett, Joseph Paul Cohen, Brian T. Layden, Perry J. Pickhardt, William Galanter

**Affiliations:** 1 Duly Health and Care, Department of Radiology, Downers Grove, IL USA; 2 Department of Biomedical and Health Information Sciences, University of Illinois Chicago, Chicago, IL USA; 3 Department of Radiology, University of Central Florida, Orlando, FL USA; 4 Brainnet, Inc., West Harrison, NY USA; 5 Department of Radiology, Emory University, Atlanta, GA USA; 6 Department of Radiology, Florida State University, Tallahassee, FL USA; 7 Department of Cardiology, Duly Health and Care, Downers Grove, IL USA; 8 EPAM, Inc, Newtown, PA USA; 9 Department of Radiology, Northwestern University, Chicago, IL USA; 10 Department of Biomedical and Health Information Sciences, UCSF, San Francisco, CA USA; 11 Center for Artificial Intelligence in Medicine, Stanford University, Stanford, CA USA; 12 Microsoft, Microsoft Corporation, Redmond, USA; 13 Department of Neurology, The University of Texas Medical Branch, Galveston, TX USA; 14 Department of Computer Science, Stanford University, Stanford, CA USA; 15 Department of Radiology, Thomas Jefferson University, Philadelphia, PA USA; 16 Bunkerhill, Palo Alto, CA USA; 17 Department of Radiology, University Hospitals Cleveland Medical Center, Cleveland, OH USA; 18 Department of Radiology, University of Wisconsin, Madison, WI USA; 19 Department of Medicine, University of Illinois Chicago, Chicago, IL USA

**Keywords:** Predictive markers, Type 2 diabetes, Prognostic markers

Correction to: *Nature Communications* 10.1038/s41467-023-39631-x, published online 07 July 2023

The original version of this Article contained an error in the Introduction section, which had the repeated sentence “They can produce useful imaging biomarkers for future medical expenses, health disparities, and multiple comorbidities”.

In addition, references 9,10,11 cited following the sentence were incorrect. These errors have now been corrected in both the PDF and HTML versions of the Article.

