# Supplementary material for: Opportunistic detection of type 2 diabetes using deep learning from frontal chest radiographs
Source: Nat Commun. 2023 Jul 7;14:4039. doi: 10.1038/s41467-023-39631-x (PMC10328953; doi:10.1038/s41467-023-39631-x)
Supplement: Supplementary file 1 — Supplementary Information [file 41467_2023_39631_MOESM1_ESM.docx]

Opportunistic Detection of Type 2 Diabetes using Deep Learning from Frontal Chest Radiographs

**Supplementary information**

**Supplementary Table 1.** Patient Demographics for Training and Validation Development Datasets 271,065 Chest Radiographs Cases*

| T2D Status | No. of Radiographic Cases | Mean Age ± SD** | Men (%) | Race/ethnicity (%) |
| --- | --- | --- | --- | --- |
| Positive | 45,961 | 66.7 ± 12.7 | 52.8 | Asian: 8  Black: 8.1  Hispanic: 7.5  Other/Unknown: 6.4  White: 71 |
| Negative | 225,104 | 57.3 ± 17.8 | 42.8 | Asian: 6  Black: 4.8  Hispanic: 6  Other/Unknown: 6.2  White: 77 |

*Radiographic cases refer to unique accessions, which consist of at least one frontal radiograph. In some cases, the frontal radiograph was repeated, often to improve patient positioning, resulting in a total of 303,604 frontal CXR images. These additional images were included in the training and validation datasets.

**Mean age of patient at the time of the chest radiograph for 160,244 distinct patients.

**Supplementary Table 2.** K-fold Cohort (Out-of-fold Predictions) Patient Characteristics (2010-2021)

|  | N. 153,168 (%) | | |
| --- | --- | --- | --- |
| Characteristics^a^ | No Diabetes  (N = 123,748) (80) | Diabetes  (N = 23,923) (16) | Poorly controlled Diabetes  (N = 5,497) (4) |
| Age, mean (SD), y | 53.3 (17.8) | 66.3 (12.7) | 59.7 (12.3) |
| Sex |  |  |  |
| Women | 69,889 (56.5) | 11,896 (49.7) | 3,150 (57.3) |
| Men | 53,859 (43.5) | 12,027 (50.3) | 2,347 (42.7) |
| Self reported  Race/Ethnicity |  | | |
| White, Non- Hispanic | 93,749 (75.8) | 17,156 (71.7) | 3,455 (62.9) |
| Black, Non- Hispanic | 6,083 (4.9) | 1,826 (7.6) | 614 (11.2) |
| Asian, Non- Hispanic | 7,315 (5.9) | 1,935 (8.1) | 406 (7.4) |
| Hispanic | 7,971 (6.4) | 1,659 (6.9) | 657 (12) |
| Other/Unknown | 8,630 (7) | 1,347 (5.6) | 365 (6.6) |
| BMI, mean (SD)^b^ | 28.6 (6.5) | 32.4 (7.3) | 34.3 (7.4) |
| SDI, mean (SD)^c^ | 24.7 (22.6) | 28.3 (24.6) | 31.4 (26.5) |
| A1c, mean (SD)^d^ | 5.49 (0.38) | 6.48 (0.78) | 8.5 (1.9) |

^a^ Data are given as number (percentage) for each group, unless specified.

^b^ N due to missingness = 871.

^c^ N due to missingness = 123.

^d^ N due to missingness = 83,111.

Source data are provided as a Source Data file.

**Supplementary Table 3.** CXR DL vs Clinical LR Performance from k-fold Cohort (Out-of-fold Predictions)

| No. | Parameters | | No. of Radiographs  (cases and controls) | | AUC (95% CI) | Prevalence (%) | NPV^1^ | PPV^1^ | Sensitivity | Specificity | F1 Score | *P*^2^ |
| --- | --- | --- | --- | --- | --- | --- | --- | --- | --- | --- | --- | --- |
| 1 | All T2D vs NoT2D | CXR DL | 29,420 | 123,748 | 0.83  (0.82, 0.83) | 19 | 0.93 | 0.39 | 0.79 | 0.71 | 0.52 | 2.2x10^-16^ |
|  |  | Clinical LR* | 29,343 | 122,835 | 0.79  (0.78, 0.79) | 19 | 0.93 | 0.34 | 0.80 | 0.63 | 0.48 |  |
| 2 | Poorly Controlled T2D vs all remaining cases and controls | CXR DL | 5,497 | 147,671 | 0.82  (0.81, 0.82) | 3.6 | 0.99 | 0.09 | 0.78 | 0.70 | 0.16 | 2.2x10^-16^ |
|  |  | Clinical LR | 5,491 | 146,687 | 0.76  (0.75, 0.76) | 3.6 | 0.99 | 0.07 | 0.79 | 0.59 | 0.12 |  |
| 3 | T2D vs NoT2D  (BMI <25, age 35–70 years) | CXR DL | 1,466 | 20,997 | 0.83  (0.81, 0.84) | 6.5 | 0.98 | 0.20 | 0.72 | 0.79 | 0.31 | 7.8x10^-13^ |
|  |  | Clinical LR | 1,464 | 20,981 | 0.78  (0.77, 0.79) | 6.5 | 0.97 | 0.15 | 0.71 | 0.71 | 0.24 |  |
| 4 | T2D vs NoT2D  (BMI ≥25**,** age 35–70) | CXR DL | 16,780 | 57,822 | 0.79  (0.79, 0.79) | 22.5 | 0.90 | 0.42 | 0.73 | 0.71 | 0.53 | 2.2x10^-16^ |
|  |  | Clinical LR | 16,767 | 57,777 | 0.73  (0.73, 0.74) | 22.5 | 0.89 | 0.35 | 0.73 | 0.61 | 0.48 |  |

*LR models all included: age, sex, BMI, race/ethnicity, language preference, SDI; differences in case counts between DL and LR models are due to observations being deleted due to missingness in LR models.

^1^Negative and positive predictive value, sensitivity and specificity were calculated using Youden’s index for an optimal threshold.

^2^P value, Comparison of AUC employing a two-sided approach through the DeLong method.

Source data are provided as a Source Data file.

**Supplementary Table 4.** Time-Dependent Area under the Receiver Operating Characteristic Curve from k-fold Cohort (Out-of-fold Predictions)

| Time | Cases of T2D | No T2D | Not yet diagnosed or Lost to follow-up^*^ | AUC (95% CI) |
| --- | --- | --- | --- | --- |
| 1 year | 2,331 | 115,716 | 14,280 | 0.80 (0.79, 0.81) |
| 3 years | 4,624 | 83,749 | 46,247 | 0.79 (0.78, 0.80) |
| 5 years | 5,901 | 56,181 | 73,815 | 0.79 (0.78, 0.80) |
| 10 years | 7,252 | 18,340 | 122,744 | 0.78 (0.77, 0.79) |

^*^ Category indicates patient is either pending physician visit without T2D diagnosis or has been lost to follow-up (censored).

Source data are provided as a Source Data file.

**Supplementary Table 5.** Patient Demographics for Emory External Validation Dataset

| T2D Status | No. of Radiographs | Mean Age ± SD* | Men (%) | Race/Ethnicity (%) |
| --- | --- | --- | --- | --- |
| Positive | 1,332 | 60.9 (14.7) | 48.3 | Asian: 3.5  Black: 56.8  Other: 4.7  Hispanic: 1.4  White: 33.6 |
| Negative | 3,677 | 48.5 (18.0) | 45.2 | Asian: 3.6  Black: 46.7  Other: 7.3  Hispanic: 2.5  White: 40.0 |

**Supplementary Table 6.** Emory External Validation Patient Characteristics (2020-2021)

|  | N. 5,026 (%) | | |
| --- | --- | --- | --- |
| Characteristics | No Diabetes  (N = 3,677) (0.73) | Diabetes  (N = 1,208) (0.24) | Poorly Controlled Diabetes  (N = 141) (0.03) |
| Age, mean (SD) | 48.5 (18.0) | 61.5 (14.7) | 55.6 (13.5) |
| Sex |  |  |  |
| Women | 2,020 (54.9) | 626 (51.8) | 65 (46.1) |
| Men | 1,657 (45.1) | 582 (48.2) | 76 (53.9) |
| Self reported  Race/Ethnicity |  | | |
| White, Non- Hispanic | 1,478 (40.2) | 416 (34.4) | 41 (29.1) |
| Black, Non- Hispanic | 1,10 (46.5) | 673 (55.7) | 88 (62.4) |
| Asian, Non- Hispanic | 134 (3.6) | 46 (3.8) | 2 (1.4) |
| Hispanic | 89 (2.4) | 18 (1.5) | 2 (1.4) |
| Other/Unknown | 266 (7.2) | 55 (4.6) | 8 (5.7) |
| BMI, mean (SD)^a^ | 28.8 (10.9) | 32.5 (9.7) | 34.2 (9.4) |
| A1c, mean (SD)^b^ | 5.4 (0.4) | 6.5 (1.0) | 11.4 (2.0) |

^a^N due to missingness = 1,899.

^b^N due to missingness = 2,811.

Source data are provided as a Source Data file.

**Supplementary Table 7.** CXR DL Performance from Emory External Validation

| No. | Parameters | | No. of Radiographs  (cases and controls) | | AUC (95% CI) | Prevalence (%) | NPV | PPV^1^ | Sensitivity | Specificity | F1 Score |
| --- | --- | --- | --- | --- | --- | --- | --- | --- | --- | --- | --- |
| 1 | All T2Dvs NoT2D | CXR DL | 1,349 | 3,677 | 0.77  (0.75, 0.78) | 26.8 | 0.86 | 0.49 | 0.66 | 0.75 | 0.56 |

^1^PPV and recall calculated from Youden’s index.
