# Supplementary material for: Opportunistic detection of type 2 diabetes using deep learning from frontal chest radiographs
Source: Nat Commun. 2023 Jul 7;14:4039. doi: 10.1038/s41467-023-39631-x (PMC10328953; doi:10.1038/s41467-023-39631-x)
Supplement: Supplementary file 3 — Description of Additional Supplementary Information File [file 41467_2023_39631_MOESM3_ESM.docx]

Opportunistic Detection of Type 2 Diabetes using Deep Learning from Frontal Chest Radiographs

**Description of Additional Supplementary Information**

Title: **Supplementary Movie 1.** Gifsplanation using Latent Feature Autoencoder

Description: This movie file displays a selection of random cases that use an autoencoder to adjust the input image. A color heatmap precedes the animated portion and emphasizes the areas of alteration. The model's predicted probability for Type 2 Diabetes (T2D) is represented by a number in the upper left corner. A significant factor influencing this prediction is the change in central mediastinal adiposity. High predictive values are associated with alterations in upper abdominal fat, indicated by an arrow, and an intense heatmap signal from the supraclavicular and rib attenuation.
