# Supplementary material for: Opportunistic detection of type 2 diabetes using deep learning from frontal chest radiographs
Source: Nat Commun. 2023 Jul 7;14:4039. doi: 10.1038/s41467-023-39631-x (PMC10328953; doi:10.1038/s41467-023-39631-x)
Supplement: Supplementary file 5 — Reporting Summary [file 41467_2023_39631_MOESM5_ESM.pdf]

nature portfolio

Corresponding author(s): Pyrros, A., Borstelmann SM, Zaiman Z, Thomas K, Mantravadi R, Price B, Greenstein E, Siddiqui N, Shulhan I, Hines-Shah, J, Lungren M, Rodriguez-Fernandez JM, Gichoya JW, Koyejo

Last updated by author(s): 3/17/2023

Reporting Summary

Nature Portfolio wishes to improve the reproducibility of the work that we publish. This form provides structure for consistency and transparency in reporting. For further information on Nature Portfolio policies, see our Editorial Policies and the Editorial Policy Checklist.

Statistics

For all statistical analyses, confirm that the following items are present in the figure legend, table legend, main text, or Methods section.

n/a	Confirmed	
☐	☒	The exact sample size (n) for each experimental group/condition, given as a discrete number and unit of measurement
☐	☒	A statement on whether measurements were taken from distinct samples or whether the same sample was measured repeatedly
☐	☒	The statistical test(s) used AND whether they are one- or two-sided<br>*Only common tests should be described solely by name; describe more complex techniques in the Methods section.*
☐	☒	A description of all covariates tested
☐	☒	A description of any assumptions or corrections, such as tests of normality and adjustment for multiple comparisons
☐	☒	A full description of the statistical parameters including central tendency (e.g. means) or other basic estimates (e.g. regression coefficient) AND variation (e.g. standard deviation) or associated estimates of uncertainty (e.g. confidence intervals)
☐	☒	For null hypothesis testing, the test statistic (e.g. F, t, r) with confidence intervals, effect sizes, degrees of freedom and P value noted<br>*Give P values as exact values whenever suitable.*
☐	☒	For Bayesian analysis, information on the choice of priors and Markov chain Monte Carlo settings
☐	☒	For hierarchical and complex designs, identification of the appropriate level for tests and full reporting of outcomes
☐	☒	Estimates of effect sizes (e.g. Cohen's d, Pearson's r), indicating how they were calculated

Our web collection on statistics for biologists contains articles on many of the points above.

Software and code

Policy information about availability of computer code

Data collection	The model weights data are available under restricted access due to privacy and ethical considerations, because of the model's capacity to consistently predict multiple potentially identifiable comorbidities and patient age across CXRs, access can be obtained by contacting AP, who will provide a response to inquiries within 14 days and supply necessary data use agreements. Researchers from established research institutes can request access, with the stipulation that commercial use is not permitted. The raw CXR and ICD10 data are protected and are not available due to data privacy laws. The Emory dataset (EMX) can be requested from JG, who will provide a response to inquiries within 14 days, subject to a data use agreement. Source data are provided with this paper.
Data analysis	We employed various software tools and libraries to perform our analyses. We used SQL Server Management Studio software (version 18.5) for clinical retrieval and Python (version 3.6) as the primary programming language. For deep learning tasks, we utilized PyTorch (version 1.01) and Captum (version 0.3.1) libraries. Our statistical analyses were conducted using R software (version 4.0; R Foundation for Statistical Computing, Vienna, Austria), incorporating the following packages: "survival" (version 3.2.13), "survivalROC" (version 1.0.3), and "pROC" (version 1.18.0). Additionally, we employed the Gifsplanation tool, available at https://github.com/mlmed/torchxrayvision.

For manuscripts utilizing custom algorithms or software that are central to the research but not yet described in published literature, software must be made available to editors and reviewers. We strongly encourage code deposition in a community repository (e.g. GitHub). See the Nature Portfolio guidelines for submitting code & software for further information.

Data

Policy information about availability of data

All manuscripts must include a data availability statement. This statement should provide the following information, where applicable:

- Accession codes, unique identifiers, or web links for publicly available datasets
- A description of any restrictions on data availability
- For clinical datasets or third party data, please ensure that the statement adheres to our policy

Model predictions for the prospective and retrospective datasets are available in github repo: https://github.com/apyrros/HCC-comorbidities.

Human research participants

Policy information about studies involving human research participants and Sex and Gender in Research.

Reporting on sex and gender	included in manuscript, table 3.
Population characteristics	included in manuscript, table 1.
Recruitment	Retrospective and observational prospective study design.
Ethics oversight	institutional review board (IRB) approval from both Edwards-Elmhurst (01-21-21_NHSR) and Emory (Chest x-ray - IRB0009197)

Note that full information on the approval of the study protocol must also be provided in the manuscript.

Field-specific reporting

Please select the one below that is the best fit for your research. If you are not sure, read the appropriate sections before making your selection.

☒ Life sciences ☐ Behavioural & social sciences ☐ Ecological, evolutionary & environmental sciences

For a reference copy of the document with all sections, see nature.com/documents/nr-reporting-summary-flat.pdf

Life sciences study design

All studies must disclose on these points even when the disclosure is negative.

Sample size	CXRs: 271,065 training set, 9,943 prospective test, k-fold 153,162, Emory external validation 5,026. The main sample size was determined by a power calculation.
Data exclusions	From the prospective cohort, patients who had previous CXRs in the development and training dataset were excluded (N = 8,272), resulting in a final total of 9,943 patients. It is typically considered inadvisable to test on data previously used in training, as the model may retain information about the patients. Consistent with other studies, patients with two or fewer claims for evaluation and management codes (CPT codes 99202 to 99499, N = 136) and five or fewer distinct encounter dates were excluded40,41, as they may have been treated within another healthcare system. Patients with type 1 diabetes (ICD9: 250.x1, 250.x3, and ICD10: E10.x, N = 188) and gestational diabetes (ICD9: 648.80–648.84, ICD10: 024.4x) diagnosis codes were also removed (Fig. 1) to mitigate potential confounding factors. These exclusion criteria were applied to the retrospective cohorts. Similar ICD10 criteria were used in the external Emory cohort, but did not have additional information regarding encounters (evaluation and management codes and encounter dates).
Replication	The internal and external validation sets were utilized, with all replication efforts proving successful and achieving an AUC 0.77. Emory, with coauthor Z.Z., independently conducted the external validation using a single dataset, one time. The deep learning model's weights were exported in ONNX format using a Python script, allowing it to function on the dataset from Emory. Additionally, the "gifsplanation" animations were developed by J.C., who also employed the ONNX-based weights and successfully replicated occlusion maps and model predictions.
Randomization	Prospective study was used for randomization with a power calculation to obtain statistical significance.
Blinding	Yes the investigators were blinded to group allocation.

Reporting for specific materials, systems and methods

We require information from authors about some types of materials, experimental systems and methods used in many studies. Here, indicate whether each material, system or method listed is relevant to your study. If you are not sure if a list item applies to your research, read the appropriate section before selecting a response.

Materials & experimental systems

n/a	Involved in the study
☒	☐ Antibodies
☒	☐ Eukaryotic cell lines
☒	☐ Palaeontology and archaeology
☒	☐ Animals and other organisms
☒	☐ Clinical data
☒	☐ Dual use research of concern

Methods

n/a	Involved in the study
☒	☐ ChIP-seq
☒	☐ Flow cytometry
☒	☐ MRI-based neuroimaging
