# Supplementary figures and images for: Opportunistic detection of type 2 diabetes using deep learning from frontal chest radiographs
Source: Nat Commun. 2023 Jul 7;14:4039. doi: 10.1038/s41467-023-39631-x (PMC10328953; doi:10.1038/s41467-023-39631-x)

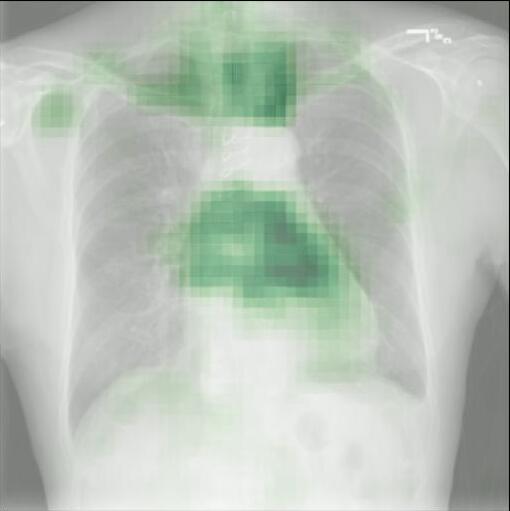

Supplement: Supplementary file 6 — Source Data [file 41467_2023_39631_MOESM6_ESM.zip › external_Emory_maps/eb0d0cbb217c77e2bd7104aa0f43c15162f97567138be97d11c6244b.pngHCC18.jpg]

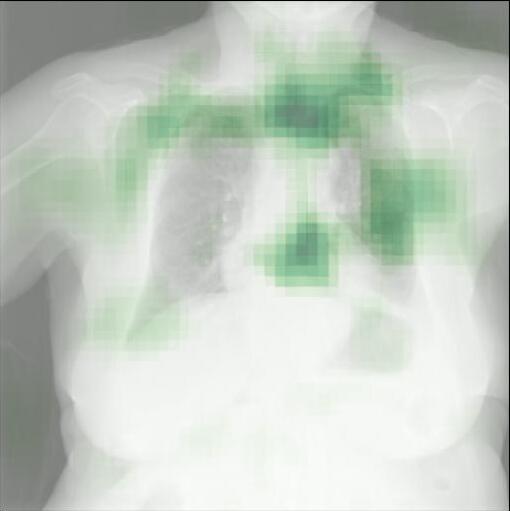

Supplement: Supplementary file 6 — Source Data [file 41467_2023_39631_MOESM6_ESM.zip › external_Emory_maps/8d06a2e76e56a1063e8cc02533dfdce4d4ae9674e6cbe9f64ea52a65.pngHCC18.jpg]

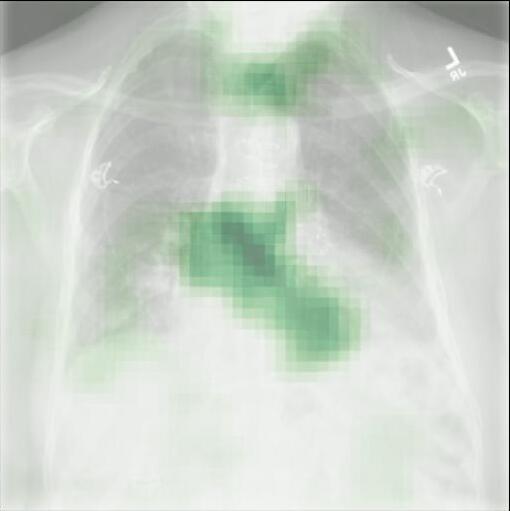

Supplement: Supplementary file 6 — Source Data [file 41467_2023_39631_MOESM6_ESM.zip › external_Emory_maps/14966aa011f2dd961e80b593df087cffeb324e2c56cfcd4ddfa3e52c.pngHCC18.jpg]

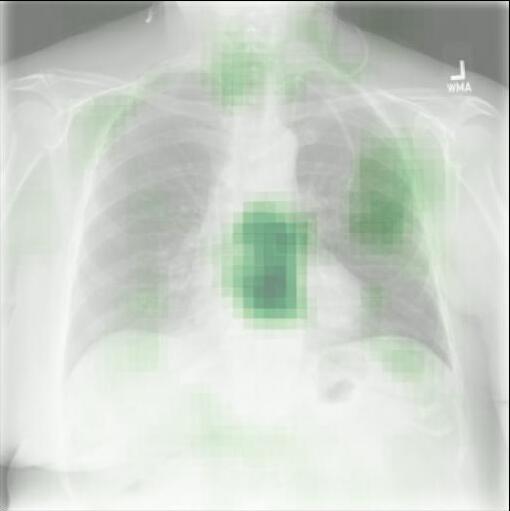

Supplement: Supplementary file 6 — Source Data [file 41467_2023_39631_MOESM6_ESM.zip › external_Emory_maps/96f2c2639a778578f67315724c7ce9602d64083e2aca3eb2671a8b0d.pngHCC18.jpg]

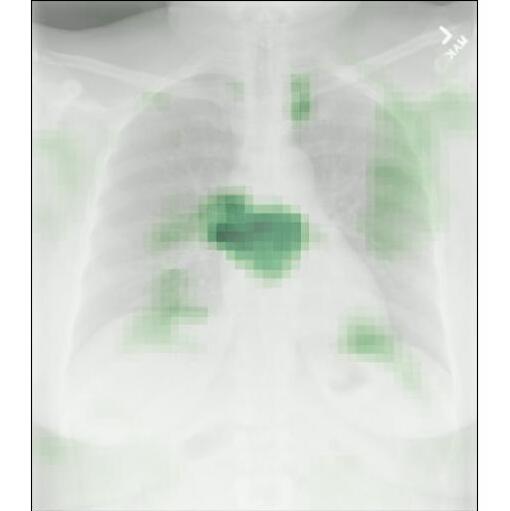

Supplement: Supplementary file 6 — Source Data [file 41467_2023_39631_MOESM6_ESM.zip › external_Emory_maps/8461ac0303e068d9ae86b8de3aa5195d380b5a1fa29899e62102afd6.pngHCC18.jpg]

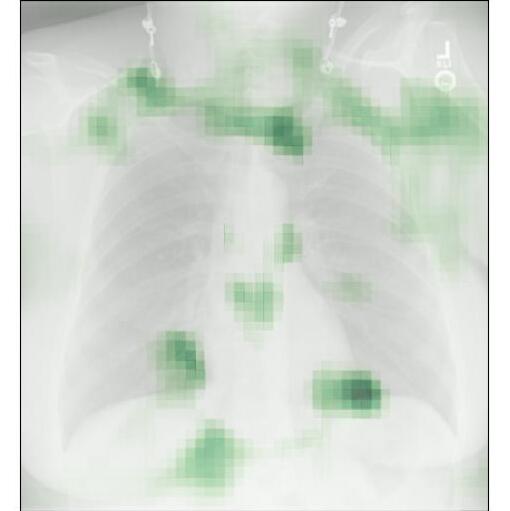

Supplement: Supplementary file 6 — Source Data [file 41467_2023_39631_MOESM6_ESM.zip › external_Emory_maps/8ef166a53391038196f085d3e0fe415d89aedc024fa6f995f12954d8.pngHCC18.jpg]

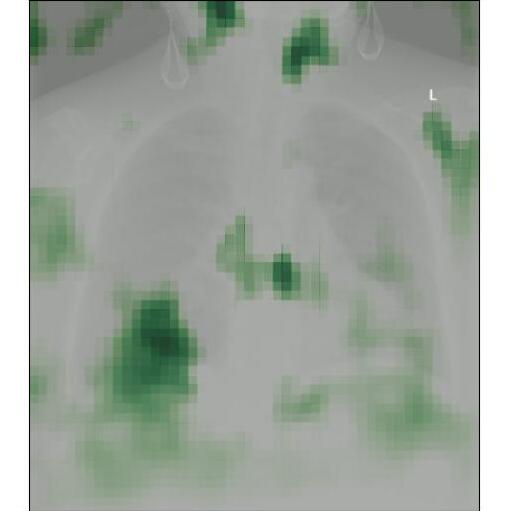

Supplement: Supplementary file 6 — Source Data [file 41467_2023_39631_MOESM6_ESM.zip › external_Emory_maps/5565e3378144b29b25aab52bbac417e4add76970b5d7f85c18f84868.pngHCC18.jpg]

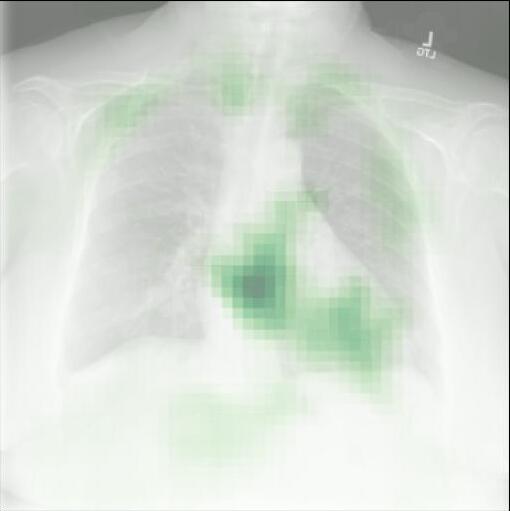

Supplement: Supplementary file 6 — Source Data [file 41467_2023_39631_MOESM6_ESM.zip › external_Emory_maps/41d7dddf407baf46582a6ab357a2081da30eee6185fa4440c6de836d.pngHCC18.jpg]

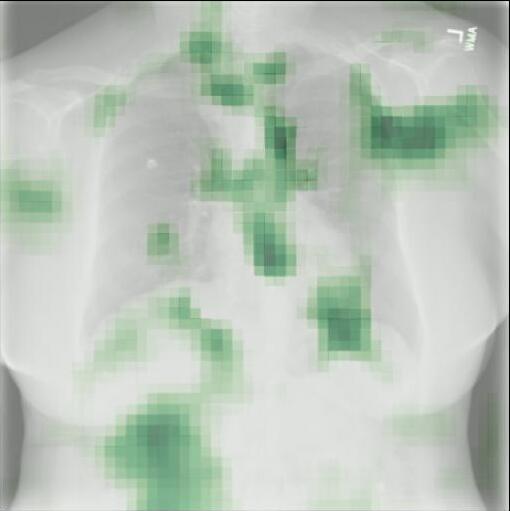

Supplement: Supplementary file 6 — Source Data [file 41467_2023_39631_MOESM6_ESM.zip › external_Emory_maps/p_8843.pngHCC18.jpg]

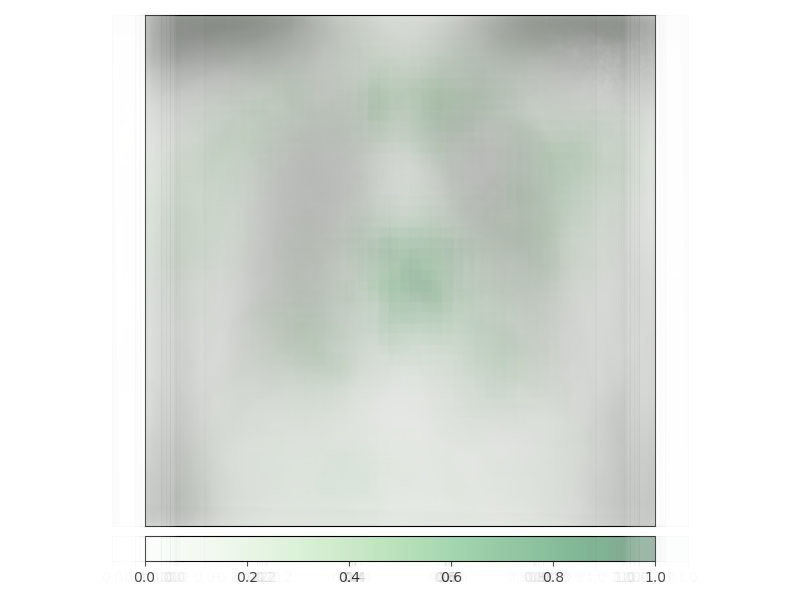

Supplement: Supplementary file 6 — Source Data [file 41467_2023_39631_MOESM6_ESM.zip › external_Emory_maps/average.png]

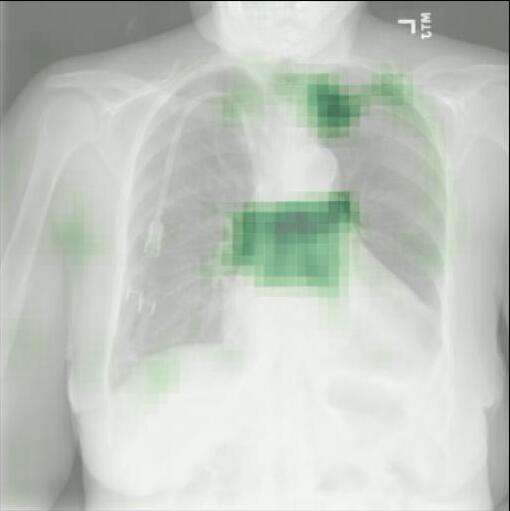

Supplement: Supplementary file 6 — Source Data [file 41467_2023_39631_MOESM6_ESM.zip › external_Emory_maps/93b774b3ce6ebf86938fabbded069c7f04a2a2ab5eb55925fd9d048b.pngHCC18.jpg]

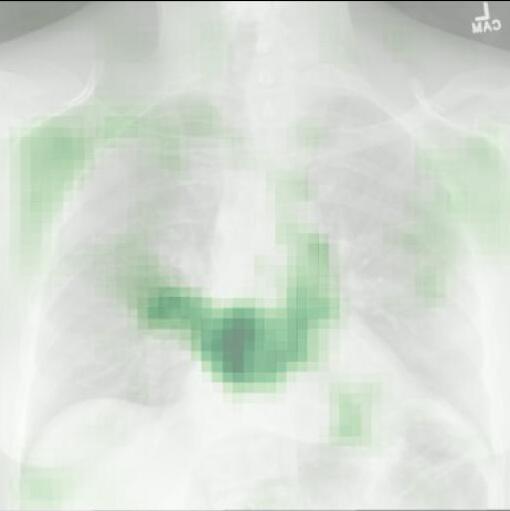

Supplement: Supplementary file 6 — Source Data [file 41467_2023_39631_MOESM6_ESM.zip › external_Emory_maps/4744045aacdc5338864c00ddf02ce002a0cccaed4070072b49d14b5c.pngHCC18.jpg]

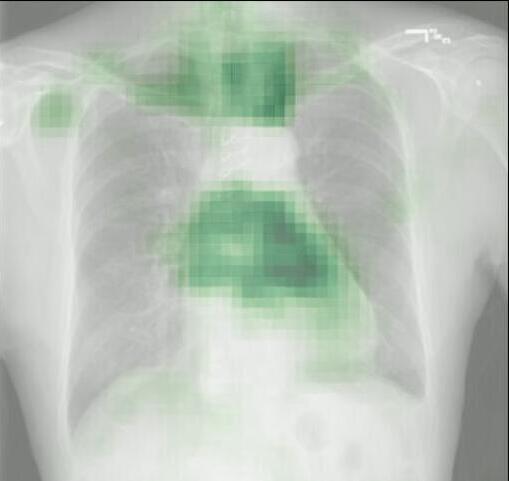

Supplement: Supplementary file 6 — Source Data [file 41467_2023_39631_MOESM6_ESM.zip › external_Emory_maps/eb0d0cbb217c77e2bd7104aa0f43c15162f97567138be97d11c6244b.pngHCC18-test.jpg]

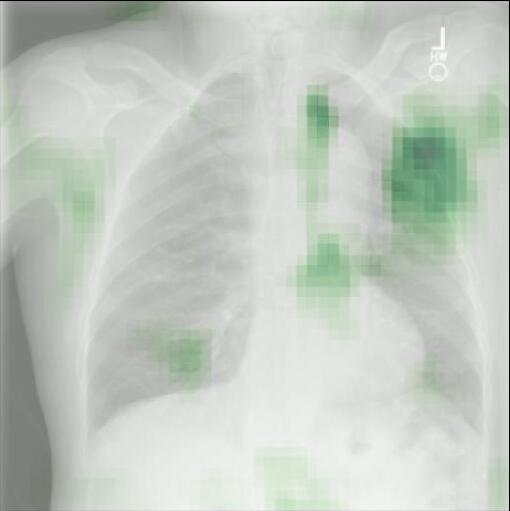

Supplement: Supplementary file 6 — Source Data [file 41467_2023_39631_MOESM6_ESM.zip › external_Emory_maps/9894be0f9303bcfa64be1f35dc808b52bc00ada6981734cc1d6919b1.pngHCC18.jpg]

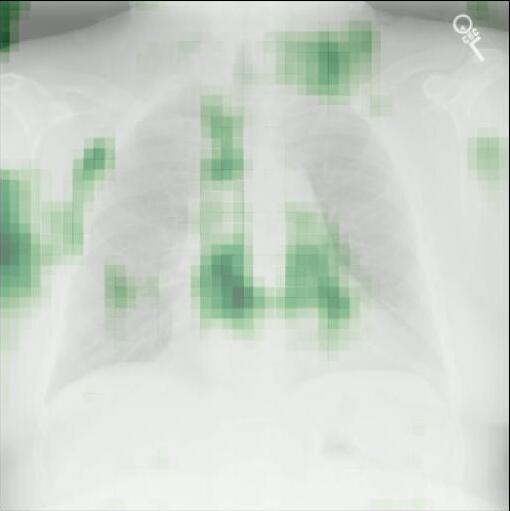

Supplement: Supplementary file 6 — Source Data [file 41467_2023_39631_MOESM6_ESM.zip › external_Emory_maps/8e06a63d7172d6d92dfc0684c161f325bbc1e075f9dd52bcde2a0d36.pngHCC18.jpg]

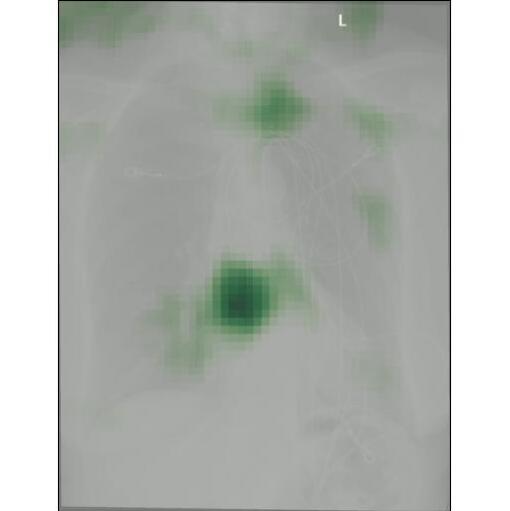

Supplement: Supplementary file 6 — Source Data [file 41467_2023_39631_MOESM6_ESM.zip › external_Emory_maps/6c688e01b92f45f94b756fcd53eaaa97f1d5beb290a53d4dd804ed09.pngHCC18.jpg]

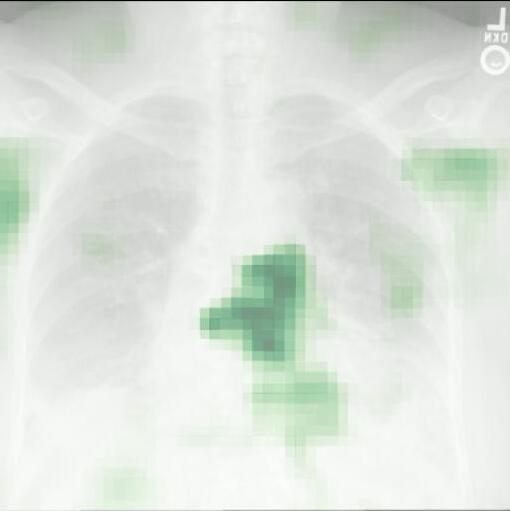

Supplement: Supplementary file 6 — Source Data [file 41467_2023_39631_MOESM6_ESM.zip › external_Emory_maps/p_18208.pngHCC18.jpg]

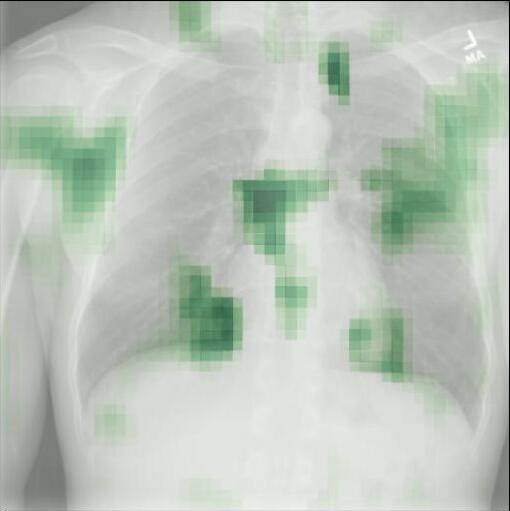

Supplement: Supplementary file 6 — Source Data [file 41467_2023_39631_MOESM6_ESM.zip › external_Emory_maps/10b9a36bc7534710beb09ba41176bf61db888bd7508987d5d8eac825.pngHCC18.jpg]

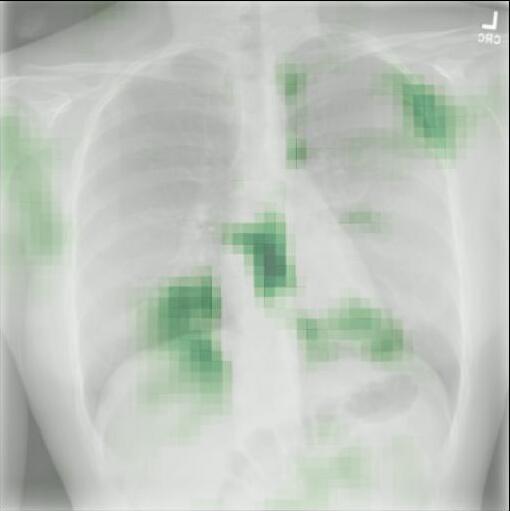

Supplement: Supplementary file 6 — Source Data [file 41467_2023_39631_MOESM6_ESM.zip › external_Emory_maps/2dc20ee5070ad89a3880902d3deeacd5f132698a9841ccb600cec17e.pngHCC18.jpg]

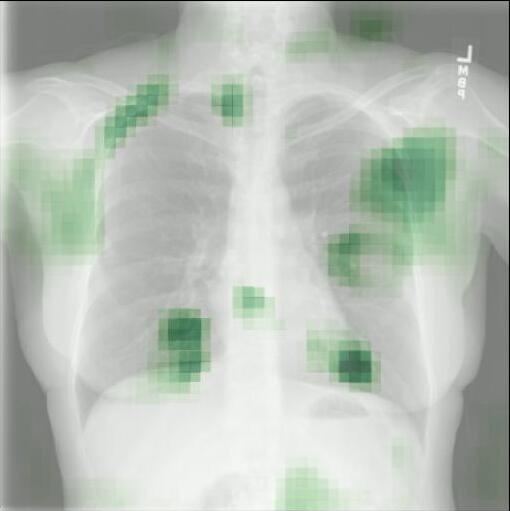

Supplement: Supplementary file 6 — Source Data [file 41467_2023_39631_MOESM6_ESM.zip › external_Emory_maps/b31507a4eab5d6ca7045a1c6f4ad380dcd050663a45761e5f53ccdd9.pngHCC18.jpg]

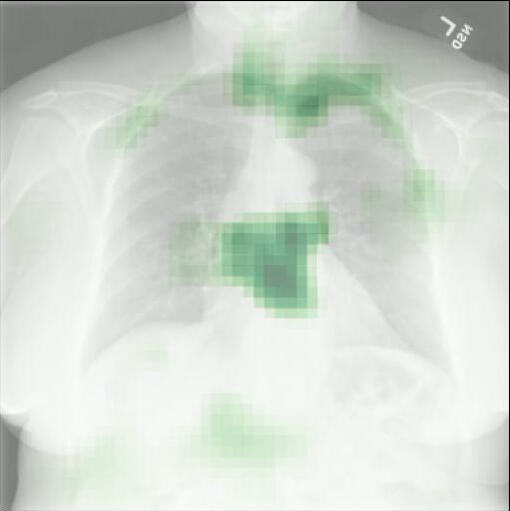

Supplement: Supplementary file 6 — Source Data [file 41467_2023_39631_MOESM6_ESM.zip › external_Emory_maps/e216ff34ef5a2adf0e9d68dae872248de916be3db41dbd15f22e9c5a.pngHCC18.jpg]

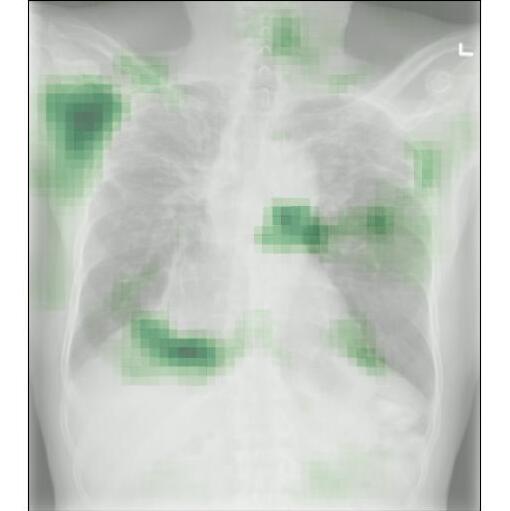

Supplement: Supplementary file 6 — Source Data [file 41467_2023_39631_MOESM6_ESM.zip › external_Emory_maps/89fc671d1714cadee072a8f329b371ee02913e6091a40b623f886098.pngHCC18.jpg]

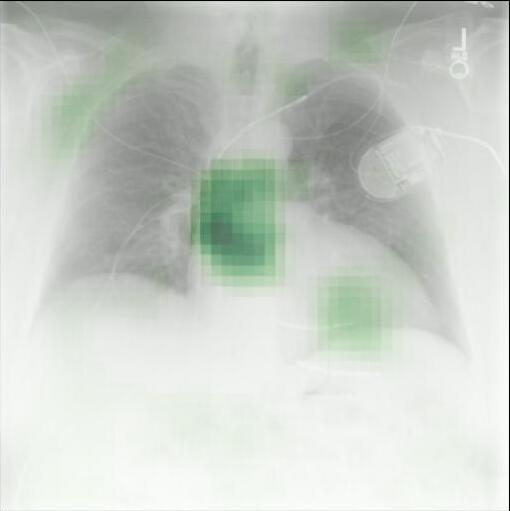

Supplement: Supplementary file 6 — Source Data [file 41467_2023_39631_MOESM6_ESM.zip › external_Emory_maps/p_40301.pngHCC18.jpg]

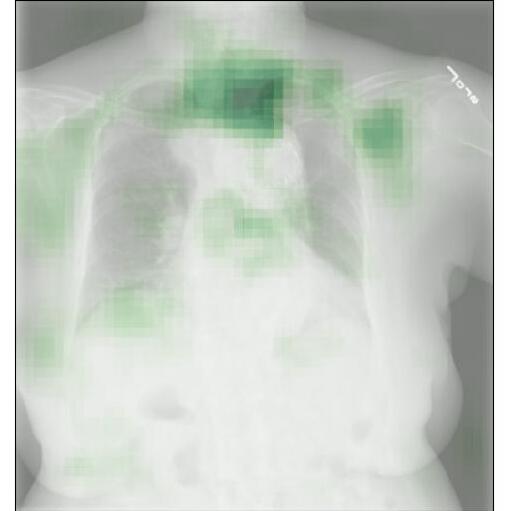

Supplement: Supplementary file 6 — Source Data [file 41467_2023_39631_MOESM6_ESM.zip › external_Emory_maps/ca46e6bae6a7ff99a073301f6c155872bb2a65ea055bd4bd7ec41485.pngHCC18.jpg]

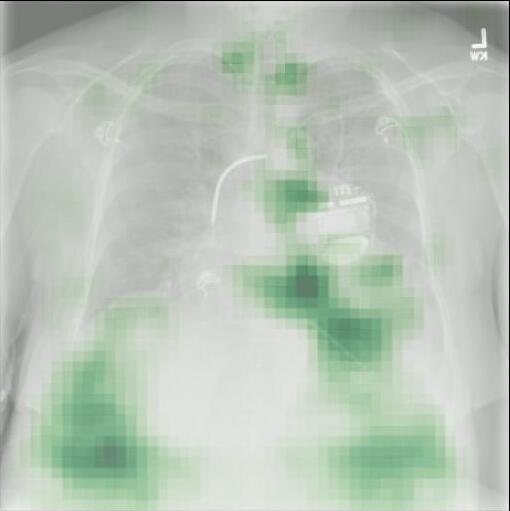

Supplement: Supplementary file 6 — Source Data [file 41467_2023_39631_MOESM6_ESM.zip › external_Emory_maps/725c006267b9241777c35729cc28edfc485377b32084e9fc67f3c0c3.pngHCC18.jpg]

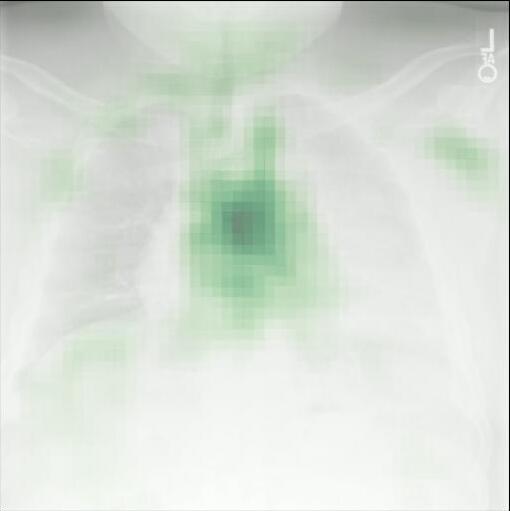

Supplement: Supplementary file 6 — Source Data [file 41467_2023_39631_MOESM6_ESM.zip › external_Emory_maps/5f7e2e980f7b58e90cf6453ff4092b14711bec3412ef9f8d0fad41b9.pngHCC18.jpg]

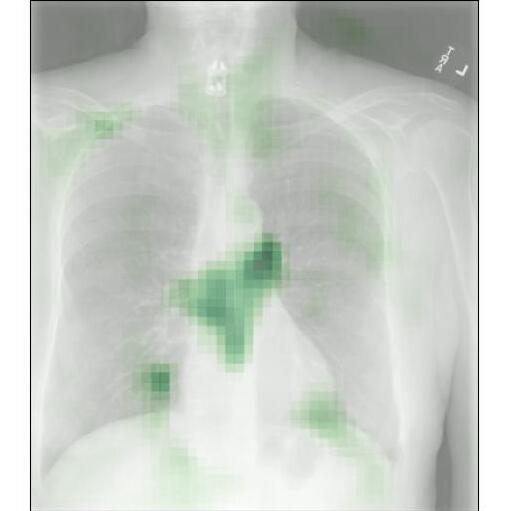

Supplement: Supplementary file 6 — Source Data [file 41467_2023_39631_MOESM6_ESM.zip › external_Emory_maps/d1fda16031a893dd4bbc3162eb5cd6899ff7a1c642b34e31813f3bdf.pngHCC18.jpg]

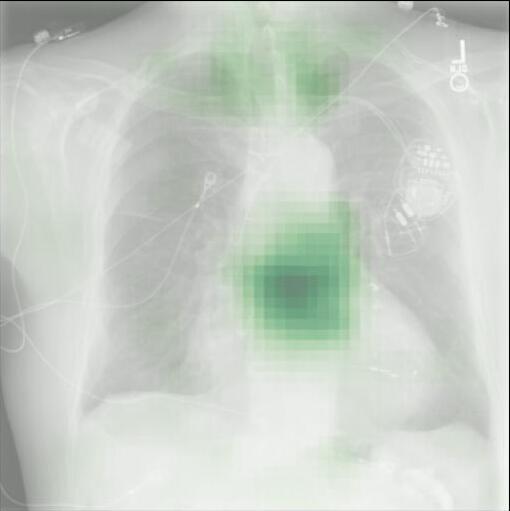

Supplement: Supplementary file 6 — Source Data [file 41467_2023_39631_MOESM6_ESM.zip › external_Emory_maps/8f62287b831d6a4d20e735354adb385ae40d0b4ffa08658f4bf82e07.pngHCC18.jpg]

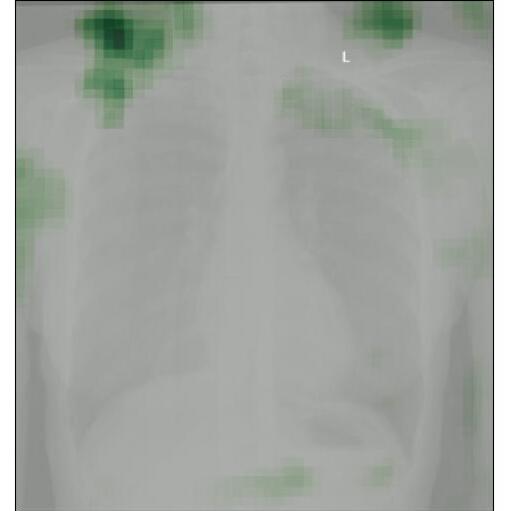

Supplement: Supplementary file 6 — Source Data [file 41467_2023_39631_MOESM6_ESM.zip › external_Emory_maps/p_34397.pngHCC18.jpg]

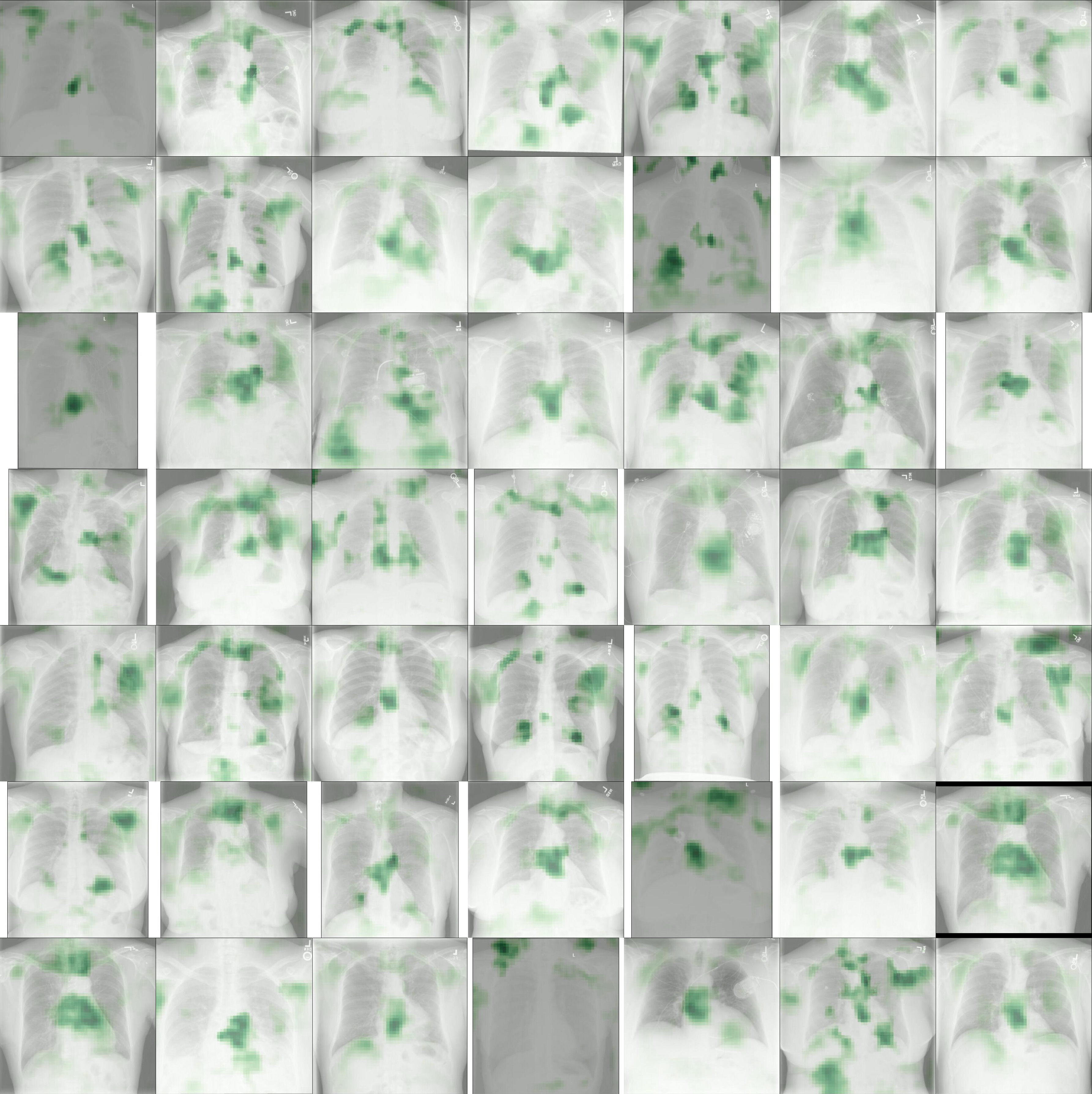

Supplement: Supplementary file 6 — Source Data [file 41467_2023_39631_MOESM6_ESM.zip › external_Emory_maps/output.jpg]

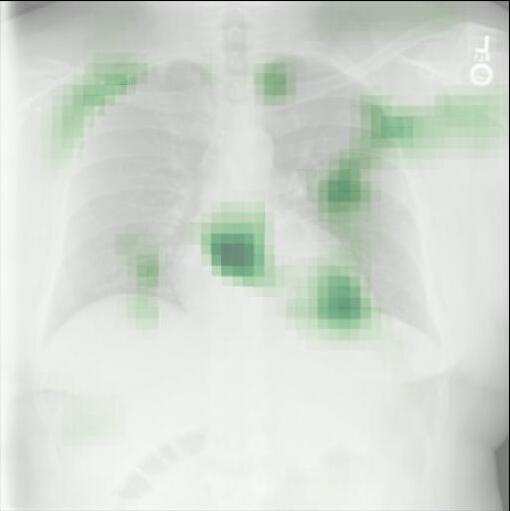

Supplement: Supplementary file 6 — Source Data [file 41467_2023_39631_MOESM6_ESM.zip › external_Emory_maps/19af4efbf8f6ab4737e4f09518c2b733358689ad808300153fa01d90.pngHCC18.jpg]

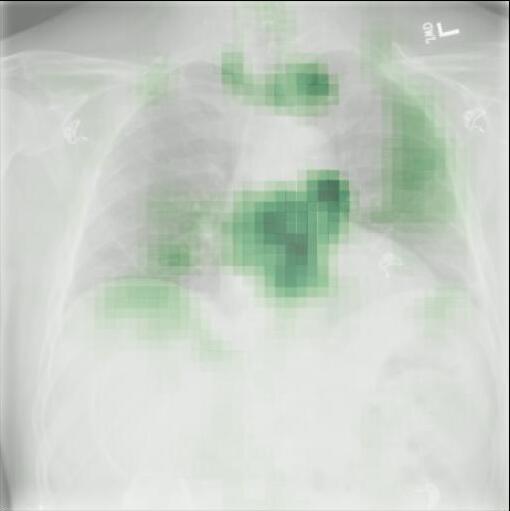

Supplement: Supplementary file 6 — Source Data [file 41467_2023_39631_MOESM6_ESM.zip › external_Emory_maps/6eb78bff3aa9303a2b54f73e271ba9ffc945aad882a8f7b92c183393.pngHCC18.jpg]

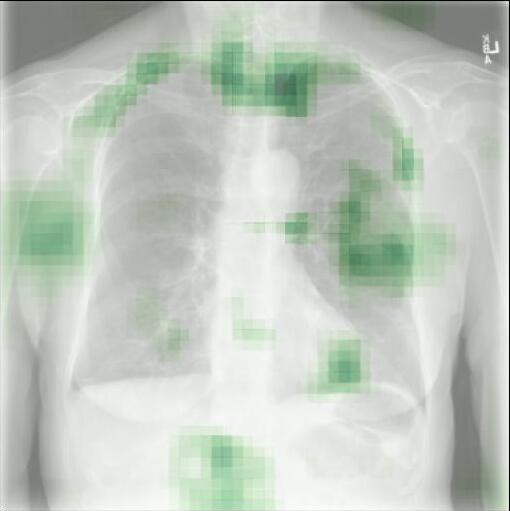

Supplement: Supplementary file 6 — Source Data [file 41467_2023_39631_MOESM6_ESM.zip › external_Emory_maps/aaace3467e112bdccd3283f1f34352efb61413757dfb5ffeecbd2de9.pngHCC18.jpg]

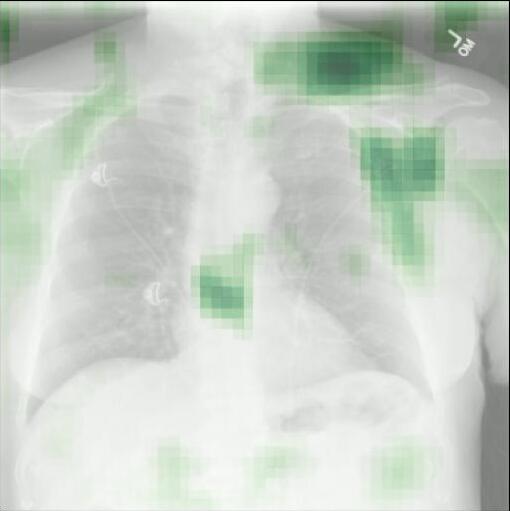

Supplement: Supplementary file 6 — Source Data [file 41467_2023_39631_MOESM6_ESM.zip › external_Emory_maps/bee52eeccc2ee2520a4f120deb157fd7e86962d1d2ea2fd472541909.pngHCC18.jpg]

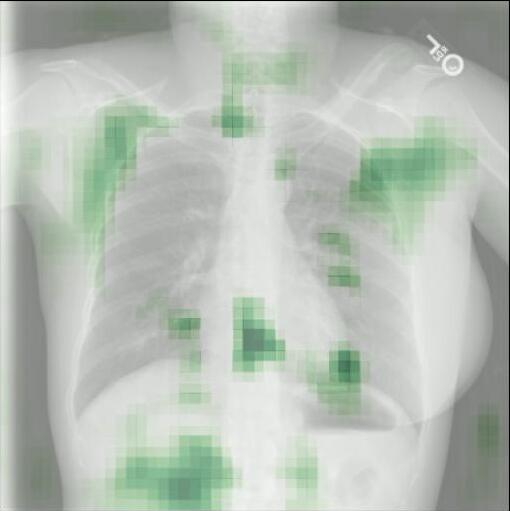

Supplement: Supplementary file 6 — Source Data [file 41467_2023_39631_MOESM6_ESM.zip › external_Emory_maps/2e8f89097ce2b0b7536eb622fa43a9f97c76e5ef08b7fd2e8ad91f9b.pngHCC18.jpg]

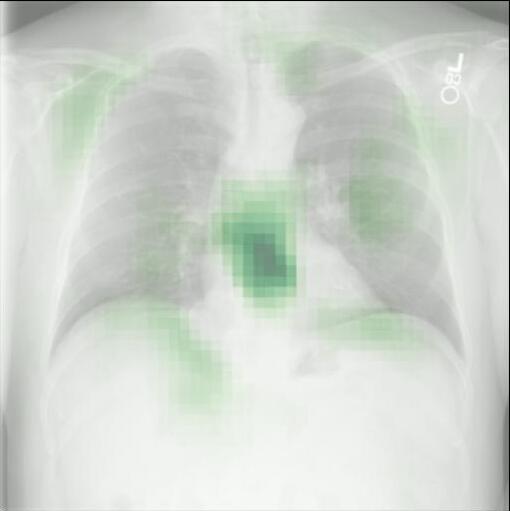

Supplement: Supplementary file 6 — Source Data [file 41467_2023_39631_MOESM6_ESM.zip › external_Emory_maps/p_9163.pngHCC18.jpg]

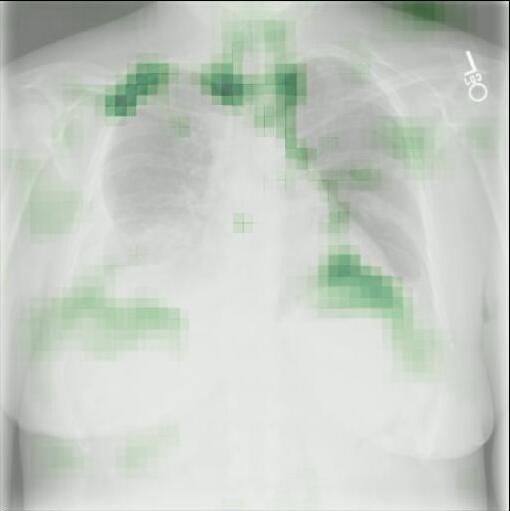

Supplement: Supplementary file 6 — Source Data [file 41467_2023_39631_MOESM6_ESM.zip › external_Emory_maps/02fa67b293010fe2ada39c4ea3bbaf5659de0f494764892812838451.pngHCC18.jpg]

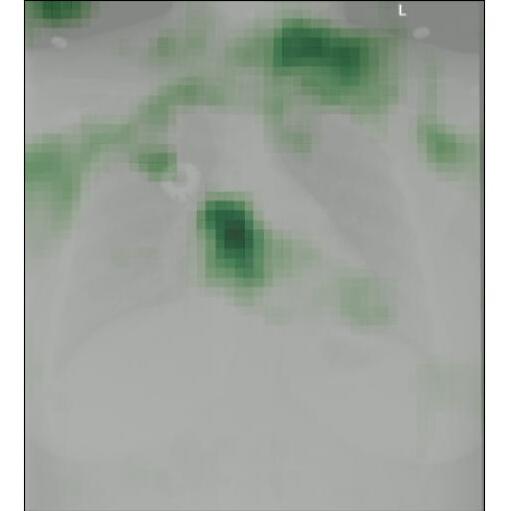

Supplement: Supplementary file 6 — Source Data [file 41467_2023_39631_MOESM6_ESM.zip › external_Emory_maps/e928051d7ccee65a496391b82fef2e3f2b15452ea9dd4f4788e1ed74.pngHCC18.jpg]

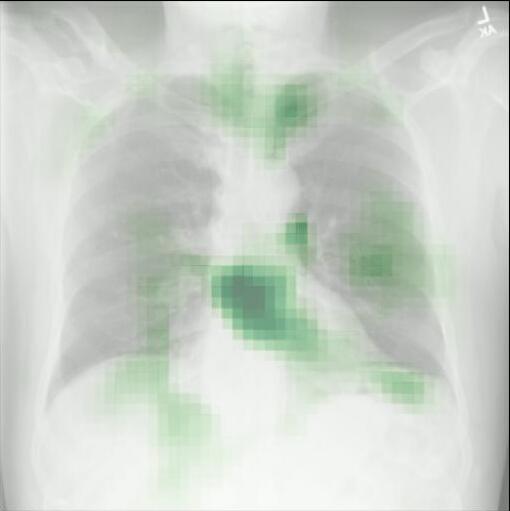

Supplement: Supplementary file 6 — Source Data [file 41467_2023_39631_MOESM6_ESM.zip › external_Emory_maps/6577a9eaa222e63da257f3360f378ced728accf7a4fe74cca53286e5.pngHCC18.jpg]

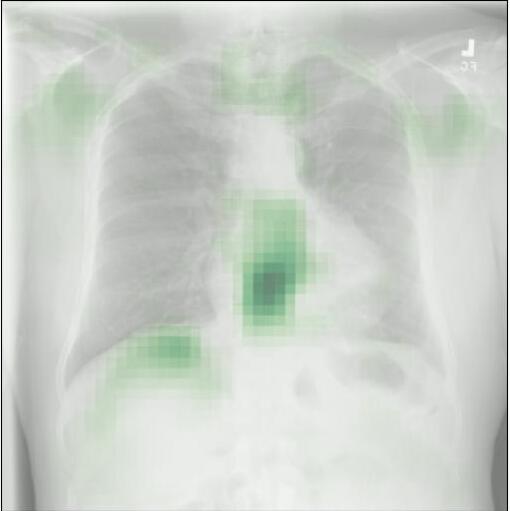

Supplement: Supplementary file 6 — Source Data [file 41467_2023_39631_MOESM6_ESM.zip › external_Emory_maps/p_18247.pngHCC18.jpg]

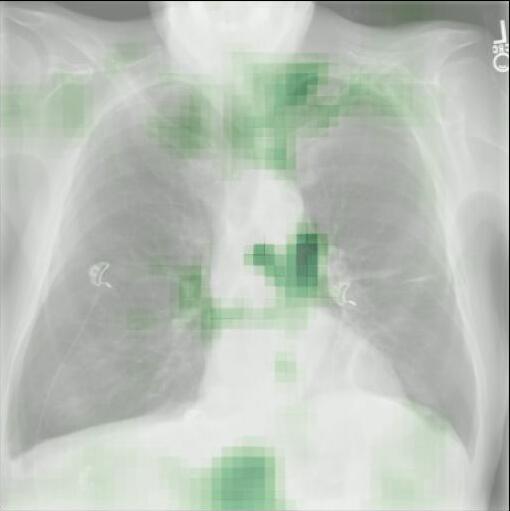

Supplement: Supplementary file 6 — Source Data [file 41467_2023_39631_MOESM6_ESM.zip › external_Emory_maps/81fcd8f12a3e97117ecdfd62952b61b197786103192e5229caa1da5b.pngHCC18.jpg]

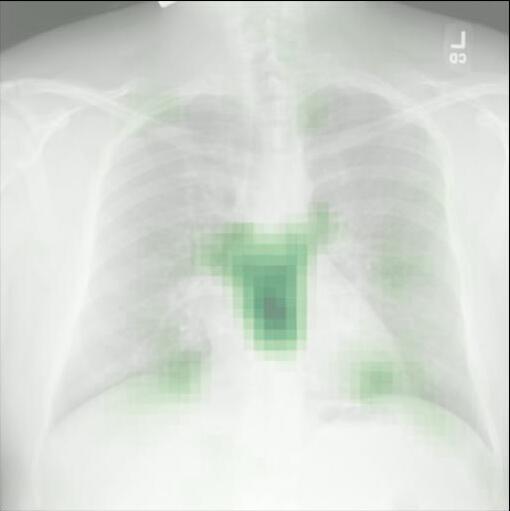

Supplement: Supplementary file 6 — Source Data [file 41467_2023_39631_MOESM6_ESM.zip › external_Emory_maps/75702982cd98199c7203d93eafbaf6874568ba5f002df90ab029eb39.pngHCC18.jpg]

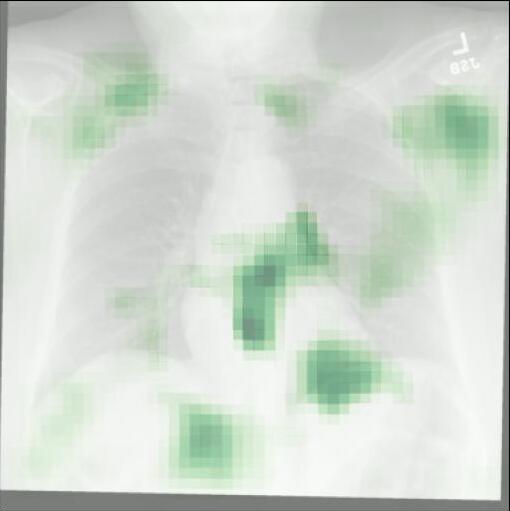

Supplement: Supplementary file 6 — Source Data [file 41467_2023_39631_MOESM6_ESM.zip › external_Emory_maps/0ce4ee737b076f387bfa2f0f0a01bdcf8f0f4b52d48e5130ab987e5e.pngHCC18.jpg]

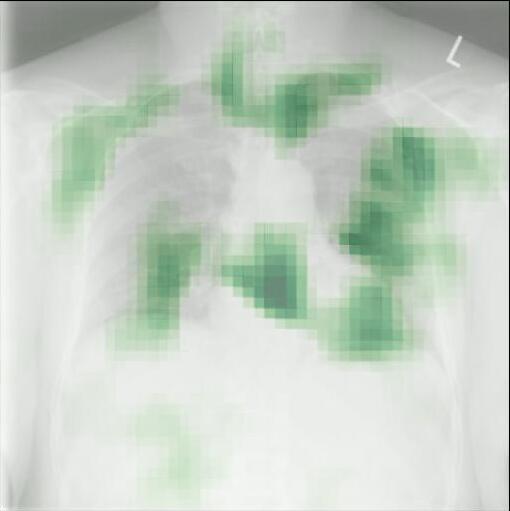

Supplement: Supplementary file 6 — Source Data [file 41467_2023_39631_MOESM6_ESM.zip › external_Emory_maps/7d4a54559625f22b58996189b78a4c5ec6d1f3763795be82b2ae5880.pngHCC18.jpg]

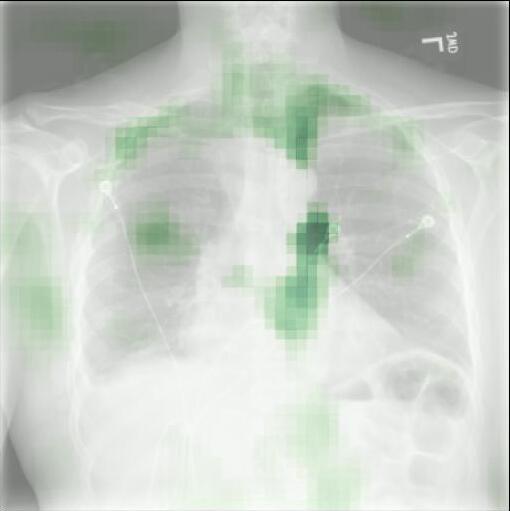

Supplement: Supplementary file 6 — Source Data [file 41467_2023_39631_MOESM6_ESM.zip › external_Emory_maps/02c395feb682ce9db1bb9237638cea52a3fc7bfb52607ba41f5af6ae.pngHCC18.jpg]

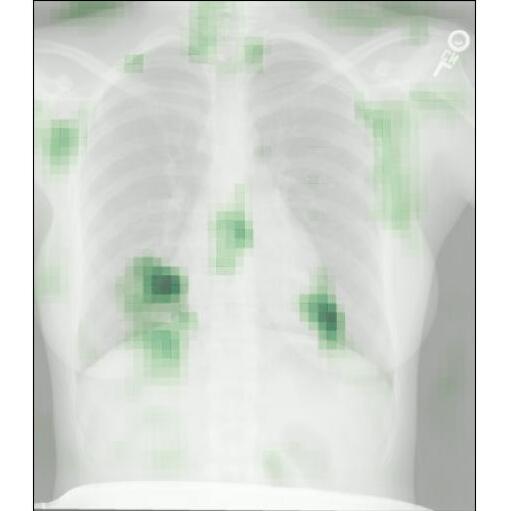

Supplement: Supplementary file 6 — Source Data [file 41467_2023_39631_MOESM6_ESM.zip › external_Emory_maps/b890233ea512d5ce74a24719042459572a744310dec540c3344a4a18.pngHCC18.jpg]

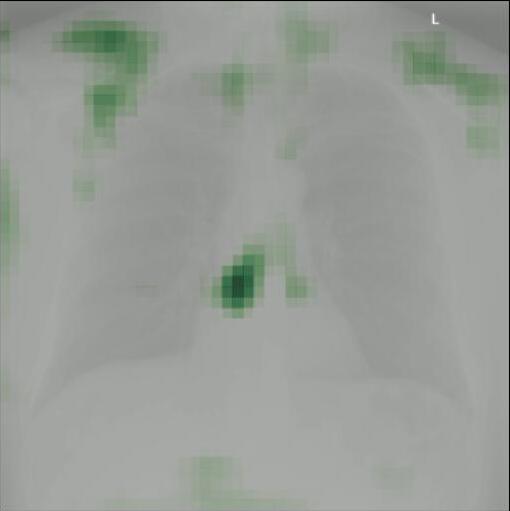

Supplement: Supplementary file 6 — Source Data [file 41467_2023_39631_MOESM6_ESM.zip › external_Emory_maps/021a26b80e7fd5010391fb696950cfcdcbb32c9204bc48cb9bccdea0.pngHCC18.jpg]

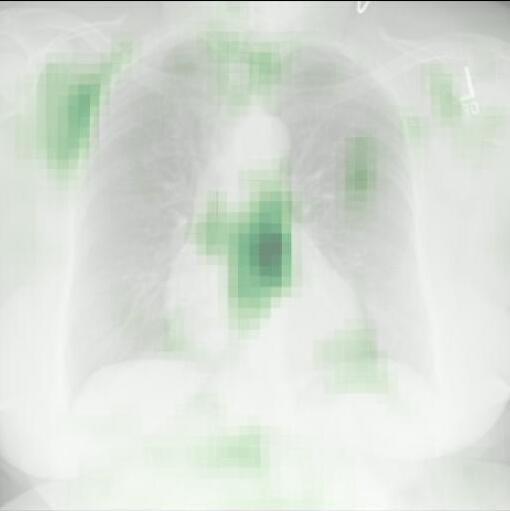

Supplement: Supplementary file 6 — Source Data [file 41467_2023_39631_MOESM6_ESM.zip › external_Emory_maps/bb9af168b85419bd1d2f5601ae4442ec743001ce78e8723586b18b33.pngHCC18.jpg]

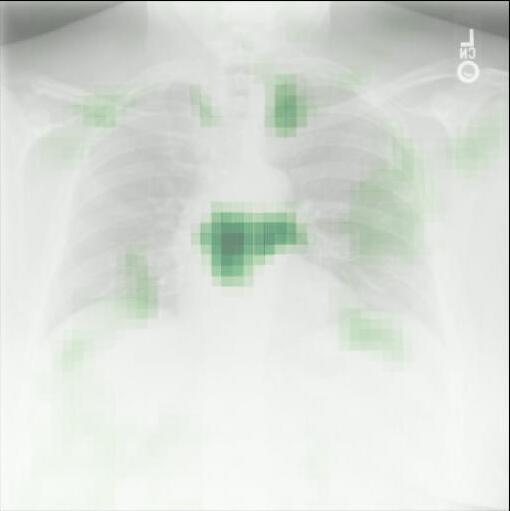

Supplement: Supplementary file 6 — Source Data [file 41467_2023_39631_MOESM6_ESM.zip › external_Emory_maps/eab4c067fa1ba586fcf5792efd41ab7ba2859019981546fd70edbccd.pngHCC18.jpg]

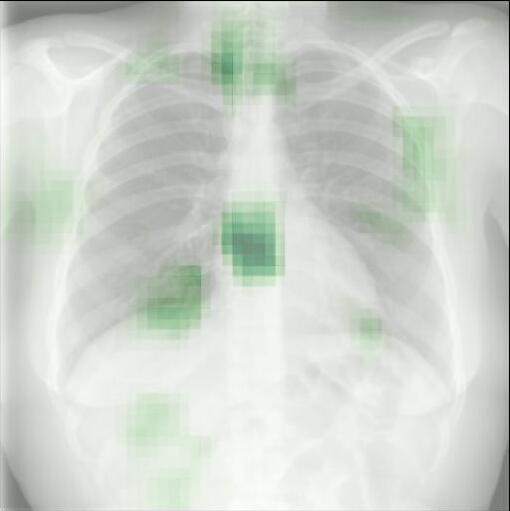

Supplement: Supplementary file 6 — Source Data [file 41467_2023_39631_MOESM6_ESM.zip › external_Emory_maps/b2ab60342c56dbec55edef53191b0704bf64ed7353659823c2238ee4.pngHCC18.jpg]

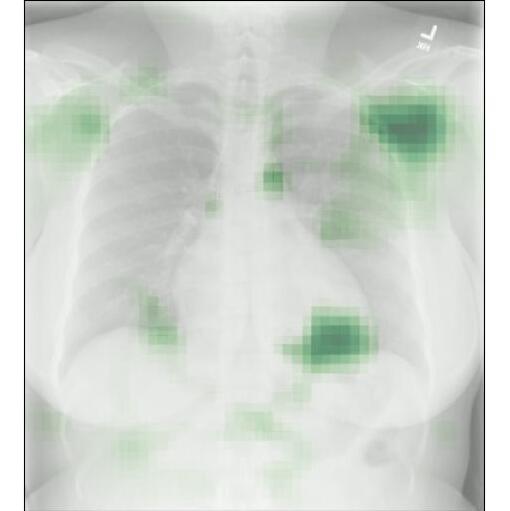

Supplement: Supplementary file 6 — Source Data [file 41467_2023_39631_MOESM6_ESM.zip › external_Emory_maps/c46e2f78e1f79c5585a2aad4324ecf3e663c46267bd54715a11878ef.pngHCC18.jpg]

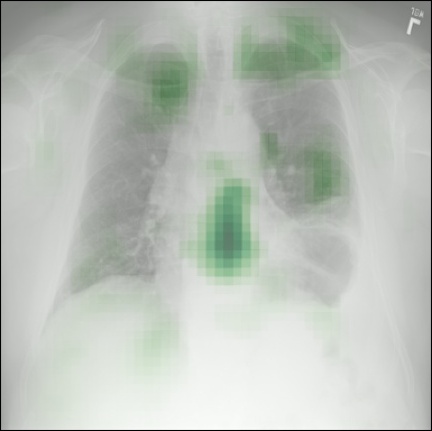

Supplement: Supplementary file 6 — Source Data [file 41467_2023_39631_MOESM6_ESM.zip › internal_maps/032311-0590_(58.0, 65.0]_151_HCC18_1_score_0.64.jpg]

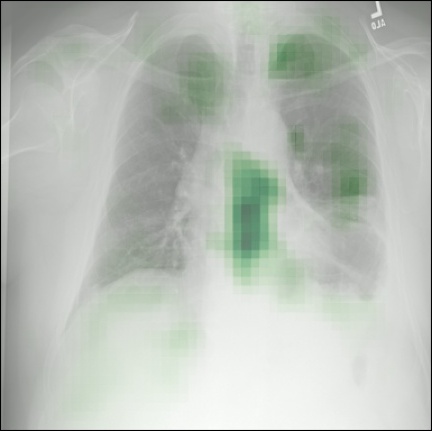

Supplement: Supplementary file 6 — Source Data [file 41467_2023_39631_MOESM6_ESM.zip › internal_maps/040611-0402_(58.0, 65.0]_153_HCC18_1_score_0.63.jpg]

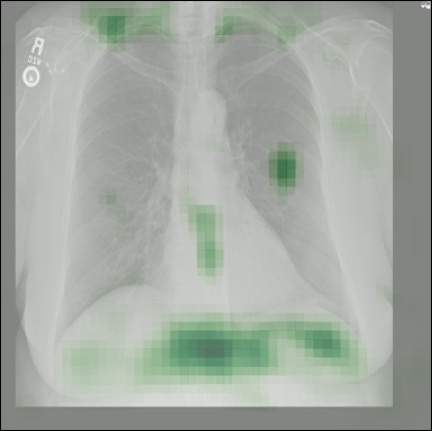

Supplement: Supplementary file 6 — Source Data [file 41467_2023_39631_MOESM6_ESM.zip › internal_maps/041514-2238_(58.0, 65.0]_139_HCC18_0_score_0.03.jpg]

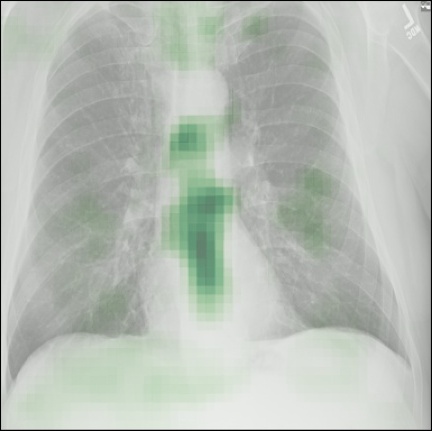

Supplement: Supplementary file 6 — Source Data [file 41467_2023_39631_MOESM6_ESM.zip › internal_maps/042518-3727_(65.0, 70.0]_60_HCC18_1_score_0.47.jpg]

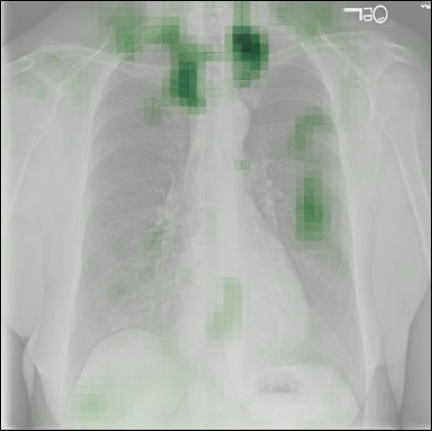

Supplement: Supplementary file 6 — Source Data [file 41467_2023_39631_MOESM6_ESM.zip › internal_maps/042516-2644_(58.0, 65.0]_168_HCC18_0_score_0.03.jpg]

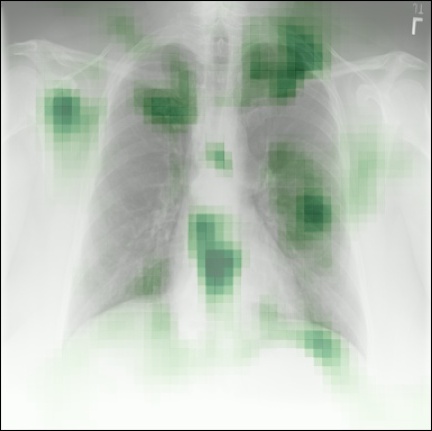

Supplement: Supplementary file 6 — Source Data [file 41467_2023_39631_MOESM6_ESM.zip › internal_maps/031512-0726_(-0.001, 58.0]_117_HCC18_1_score_0.57.jpg]

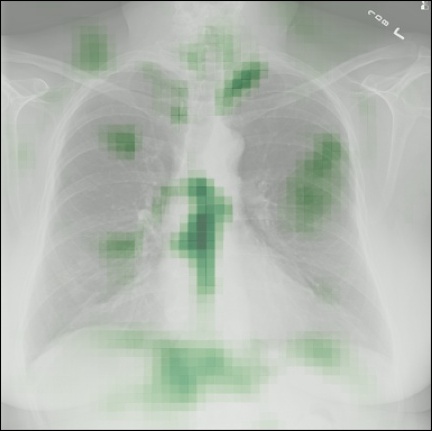

Supplement: Supplementary file 6 — Source Data [file 41467_2023_39631_MOESM6_ESM.zip › internal_maps/051718-3337_(70.0, 77.0]_202_HCC18_0_score_0.03.jpg]

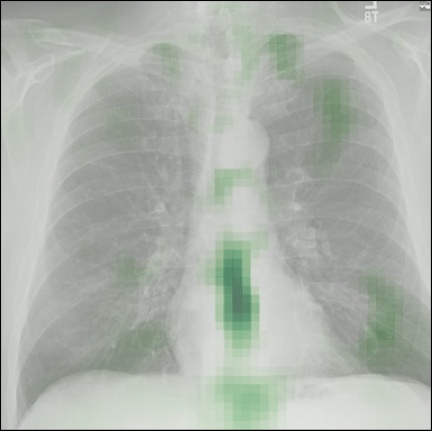

Supplement: Supplementary file 6 — Source Data [file 41467_2023_39631_MOESM6_ESM.zip › internal_maps/030819-3288_(65.0, 70.0]_53_HCC18_1_score_0.26.jpg]

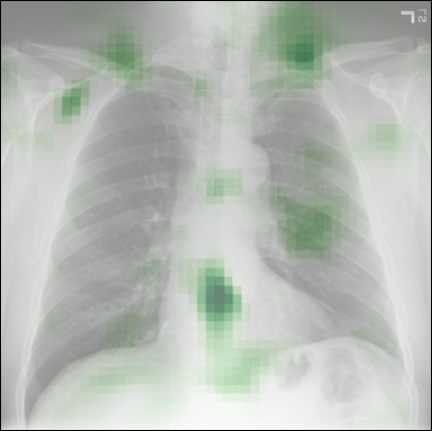

Supplement: Supplementary file 6 — Source Data [file 41467_2023_39631_MOESM6_ESM.zip › internal_maps/040313-0774_(58.0, 65.0]_95_HCC18_0_score_0.13.jpg]

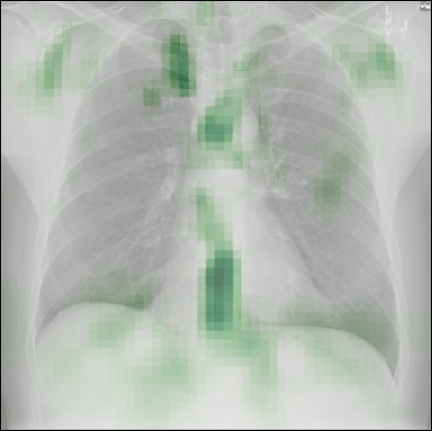

Supplement: Supplementary file 6 — Source Data [file 41467_2023_39631_MOESM6_ESM.zip › internal_maps/030821-4507_(65.0, 70.0]_66_HCC18_1_score_0.09.jpg]

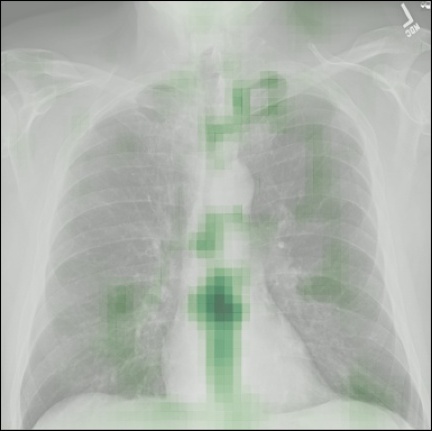

Supplement: Supplementary file 6 — Source Data [file 41467_2023_39631_MOESM6_ESM.zip › internal_maps/042518-3727_(65.0, 70.0]_59_HCC18_1_score_0.35.jpg]

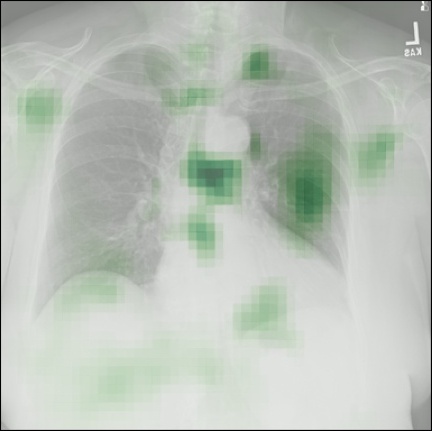

Supplement: Supplementary file 6 — Source Data [file 41467_2023_39631_MOESM6_ESM.zip › internal_maps/041619-4145_(70.0, 77.0]_42_HCC18_0_score_0.34.jpg]

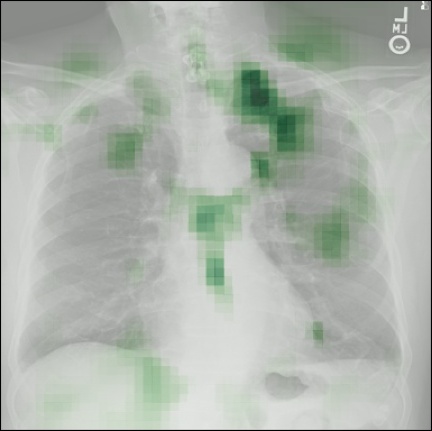

Supplement: Supplementary file 6 — Source Data [file 41467_2023_39631_MOESM6_ESM.zip › internal_maps/030519-3370_(77.0, 97.0]_43_HCC18_1_score_0.08.jpg]

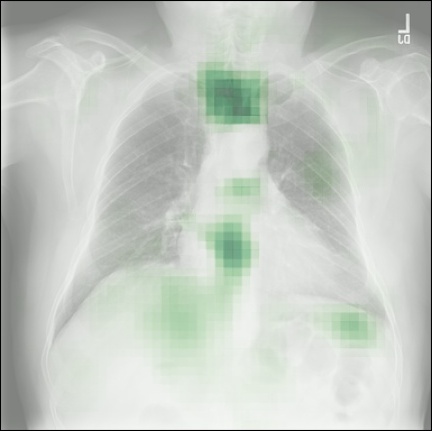

Supplement: Supplementary file 6 — Source Data [file 41467_2023_39631_MOESM6_ESM.zip › internal_maps/021221-3413_(70.0, 77.0]_163_HCC18_0_score_0.30.jpg]

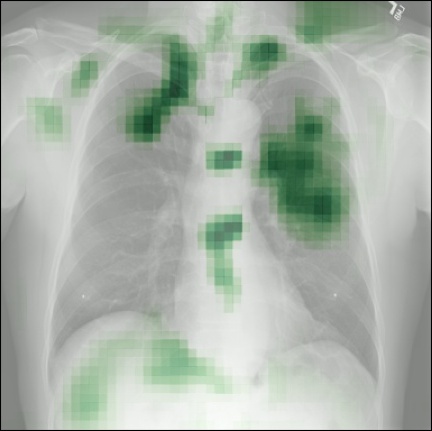

Supplement: Supplementary file 6 — Source Data [file 41467_2023_39631_MOESM6_ESM.zip › internal_maps/040611-0829_(58.0, 65.0]_190_HCC18_0_score_0.14.jpg]

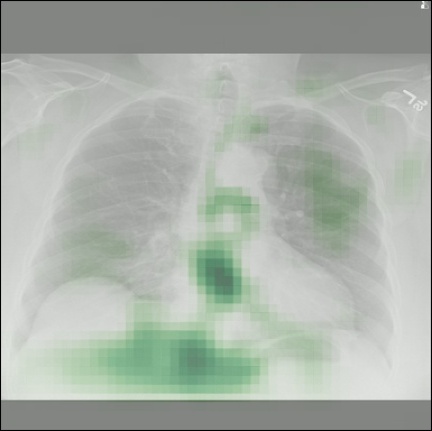

Supplement: Supplementary file 6 — Source Data [file 41467_2023_39631_MOESM6_ESM.zip › internal_maps/033018-3114_(65.0, 70.0]_5_HCC18_1_score_0.34.jpg]

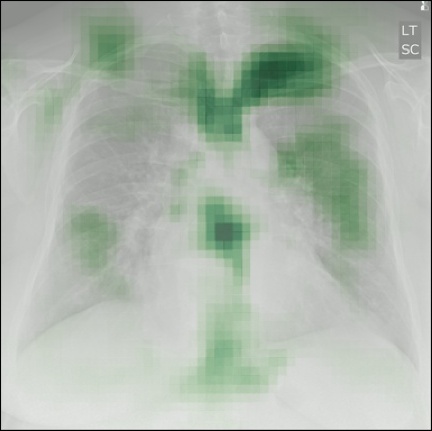

Supplement: Supplementary file 6 — Source Data [file 41467_2023_39631_MOESM6_ESM.zip › internal_maps/011314-2447_(65.0, 70.0]_174_HCC18_1_score_0.87.jpg]

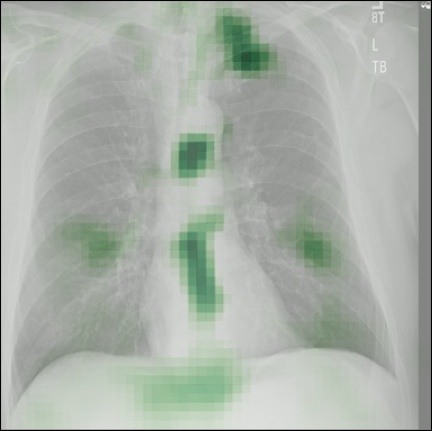

Supplement: Supplementary file 6 — Source Data [file 41467_2023_39631_MOESM6_ESM.zip › internal_maps/030819-3288_(65.0, 70.0]_54_HCC18_1_score_0.45.jpg]

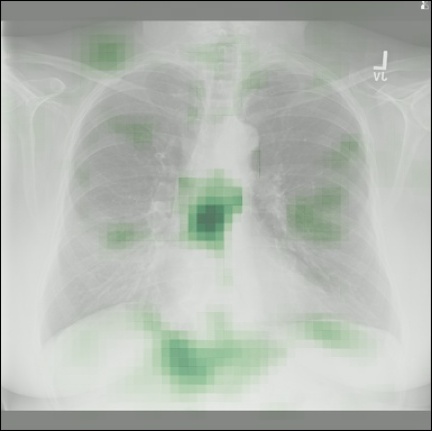

Supplement: Supplementary file 6 — Source Data [file 41467_2023_39631_MOESM6_ESM.zip › internal_maps/011520-3628_(70.0, 77.0]_198_HCC18_0_score_0.03.jpg]

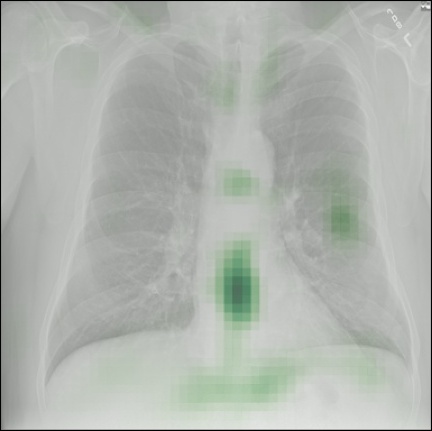

Supplement: Supplementary file 6 — Source Data [file 41467_2023_39631_MOESM6_ESM.zip › internal_maps/020118-2995_(65.0, 70.0]_0_HCC18_1_score_0.40.jpg]

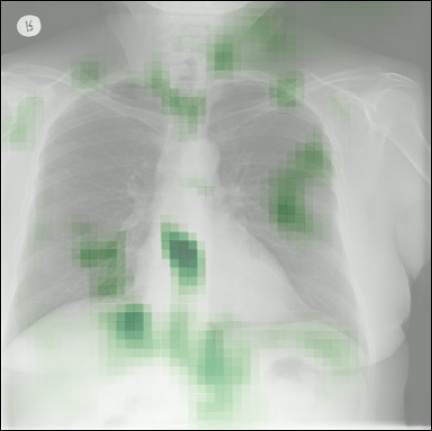

Supplement: Supplementary file 6 — Source Data [file 41467_2023_39631_MOESM6_ESM.zip › internal_maps/022210-0721_(-0.001, 58.0]_138_HCC18_0_score_0.02.jpg]

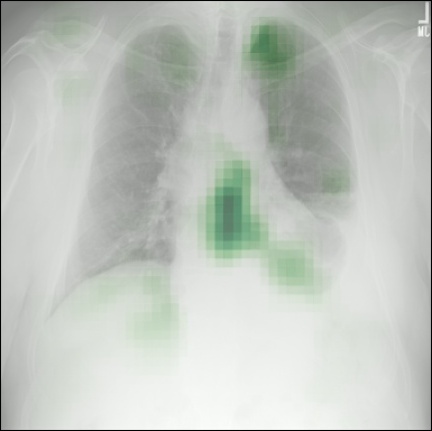

Supplement: Supplementary file 6 — Source Data [file 41467_2023_39631_MOESM6_ESM.zip › internal_maps/030211-0370_(58.0, 65.0]_149_HCC18_1_score_0.59.jpg]

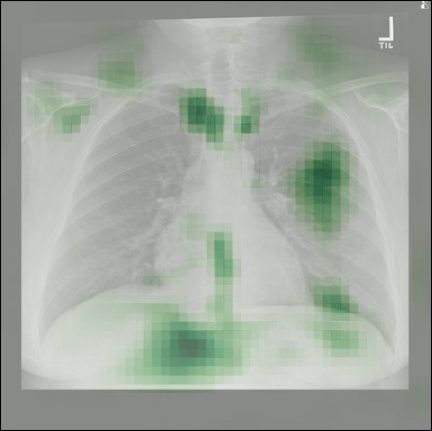

Supplement: Supplementary file 6 — Source Data [file 41467_2023_39631_MOESM6_ESM.zip › internal_maps/011817-2997_(-0.001, 58.0]_112_HCC18_1_score_0.16.jpg]

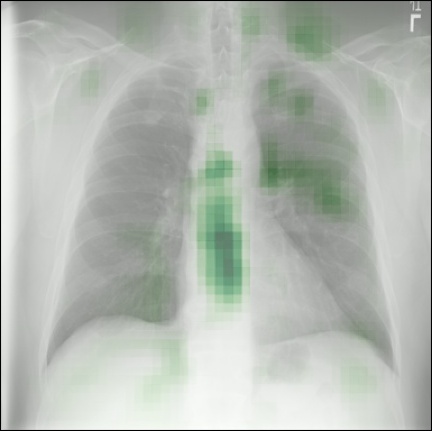

Supplement: Supplementary file 6 — Source Data [file 41467_2023_39631_MOESM6_ESM.zip › internal_maps/010710-0506_(70.0, 77.0]_194_HCC18_0_score_0.42.jpg]

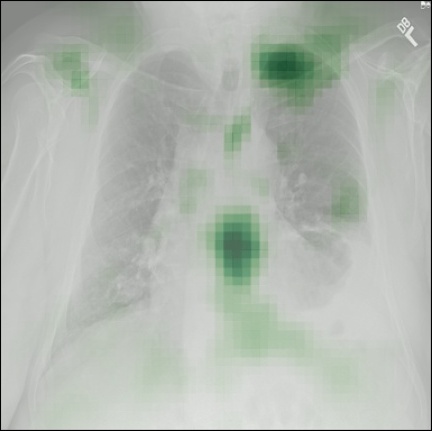

Supplement: Supplementary file 6 — Source Data [file 41467_2023_39631_MOESM6_ESM.zip › internal_maps/013115-0632_(58.0, 65.0]_148_HCC18_1_score_0.79.jpg]

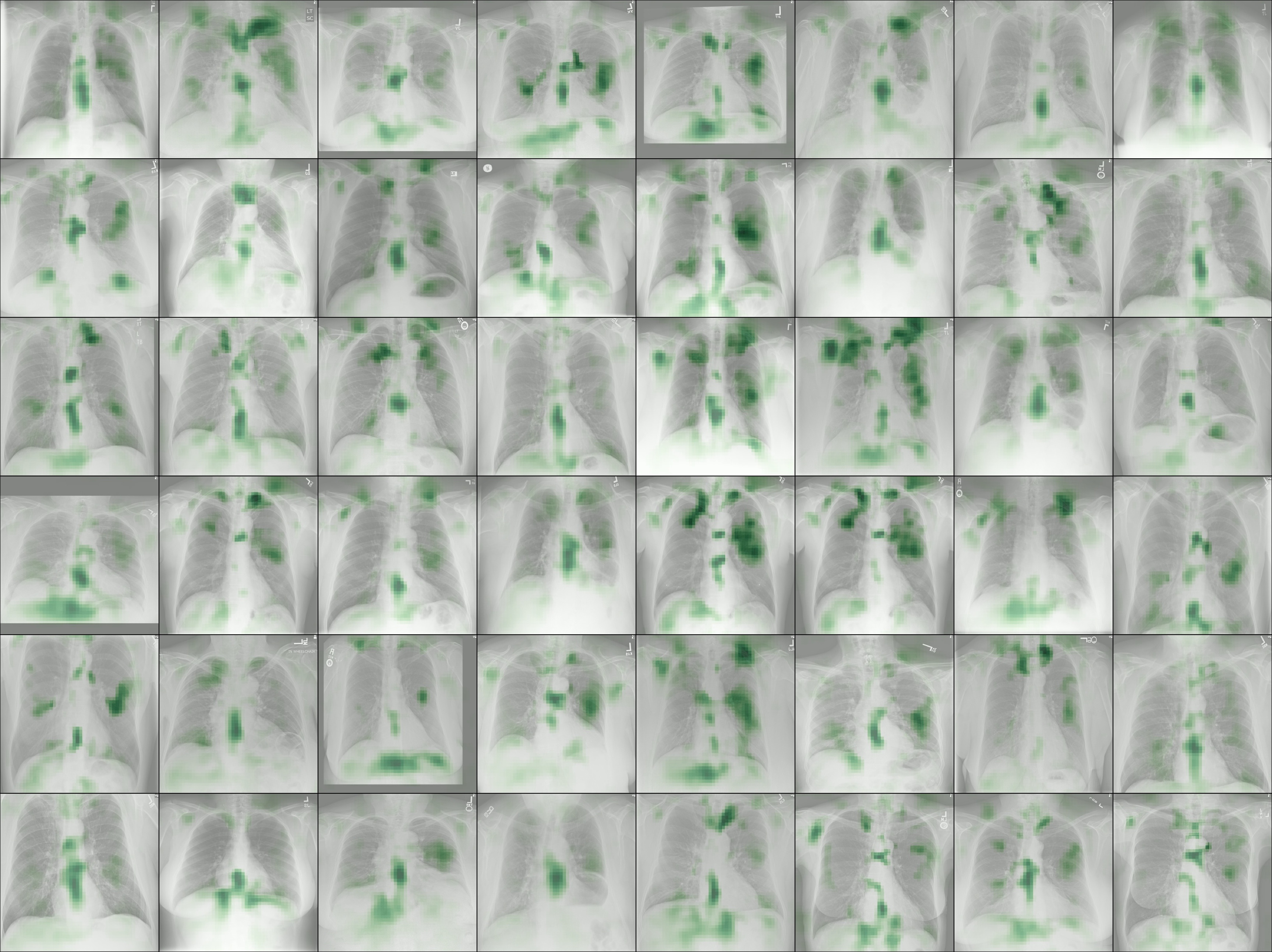

Supplement: Supplementary file 6 — Source Data [file 41467_2023_39631_MOESM6_ESM.zip › internal_maps/contactsheet.jpg]

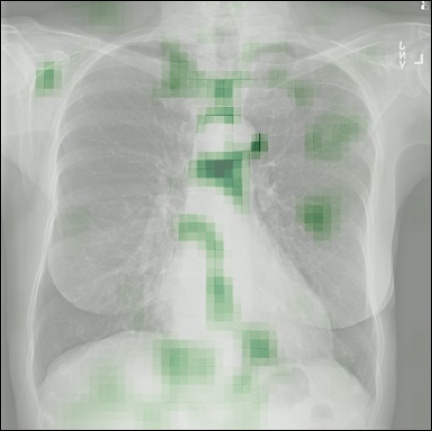

Supplement: Supplementary file 6 — Source Data [file 41467_2023_39631_MOESM6_ESM.zip › internal_maps/051921-4418_(70.0, 77.0]_37_HCC18_0_score_0.02.jpg]

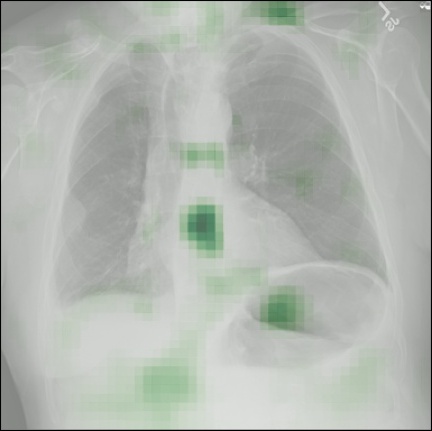

Supplement: Supplementary file 6 — Source Data [file 41467_2023_39631_MOESM6_ESM.zip › internal_maps/032719-3500_(77.0, 97.0]_28_HCC18_0_score_0.10.jpg]

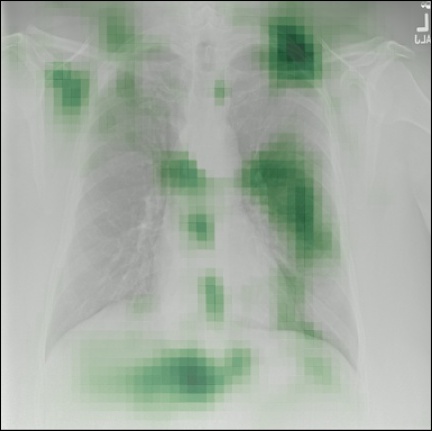

Supplement: Supplementary file 6 — Source Data [file 41467_2023_39631_MOESM6_ESM.zip › internal_maps/042015-1917_(58.0, 65.0]_121_HCC18_1_score_0.69.jpg]

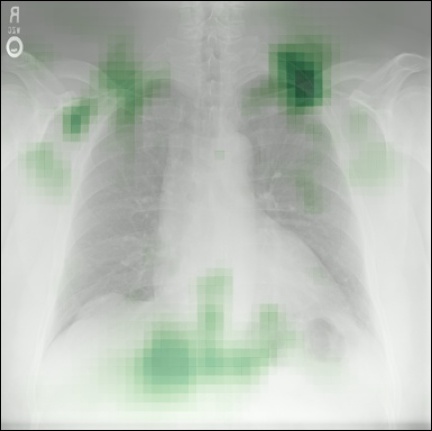

Supplement: Supplementary file 6 — Source Data [file 41467_2023_39631_MOESM6_ESM.zip › internal_maps/040711-0569_(58.0, 65.0]_129_HCC18_0_score_0.23.jpg]

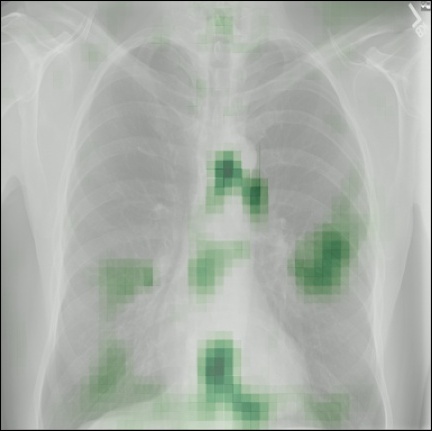

Supplement: Supplementary file 6 — Source Data [file 41467_2023_39631_MOESM6_ESM.zip › internal_maps/040720-1299_(65.0, 70.0]_132_HCC18_0_score_0.02.jpg]

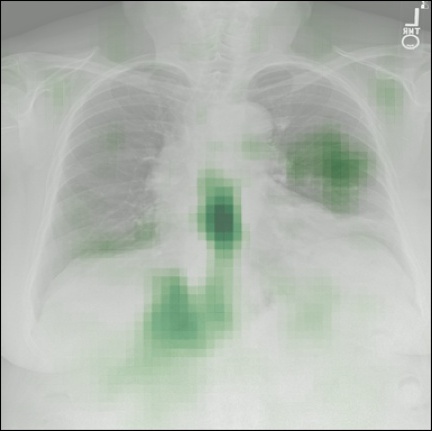

Supplement: Supplementary file 6 — Source Data [file 41467_2023_39631_MOESM6_ESM.zip › internal_maps/050216-2105_(70.0, 77.0]_24_HCC18_0_score_0.32.jpg]

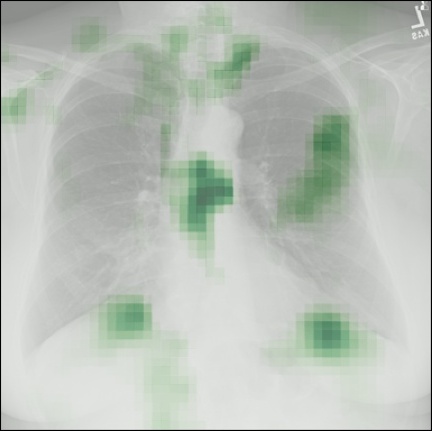

Supplement: Supplementary file 6 — Source Data [file 41467_2023_39631_MOESM6_ESM.zip › internal_maps/020720-3583_(70.0, 77.0]_200_HCC18_0_score_0.03.jpg]

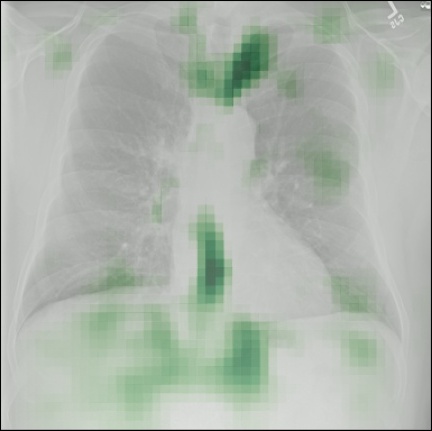

Supplement: Supplementary file 6 — Source Data [file 41467_2023_39631_MOESM6_ESM.zip › internal_maps/050918-2984_(65.0, 70.0]_3_HCC18_1_score_0.26.jpg]

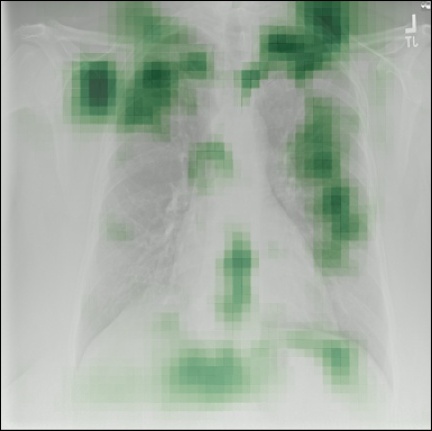

Supplement: Supplementary file 6 — Source Data [file 41467_2023_39631_MOESM6_ESM.zip › internal_maps/031715-1624_(58.0, 65.0]_120_HCC18_1_score_0.66.jpg]

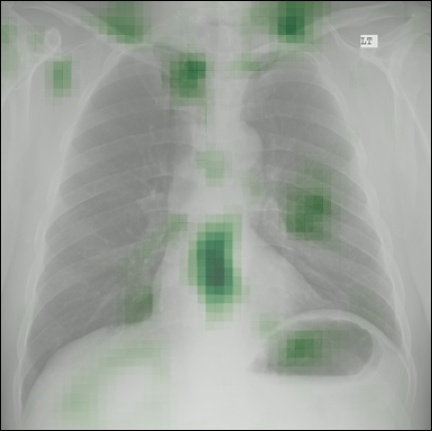

Supplement: Supplementary file 6 — Source Data [file 41467_2023_39631_MOESM6_ESM.zip › internal_maps/021510-0567_(-0.001, 58.0]_93_HCC18_0_score_0.30.jpg]

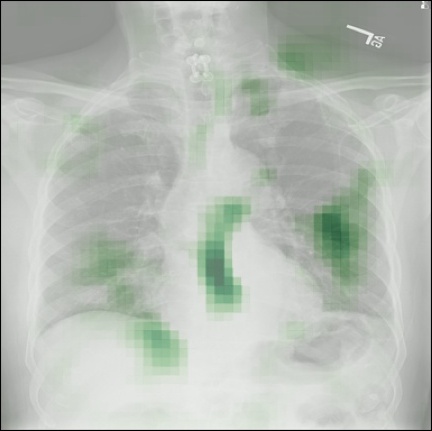

Supplement: Supplementary file 6 — Source Data [file 41467_2023_39631_MOESM6_ESM.zip › internal_maps/042018-2925_(77.0, 97.0]_46_HCC18_1_score_0.09.jpg]

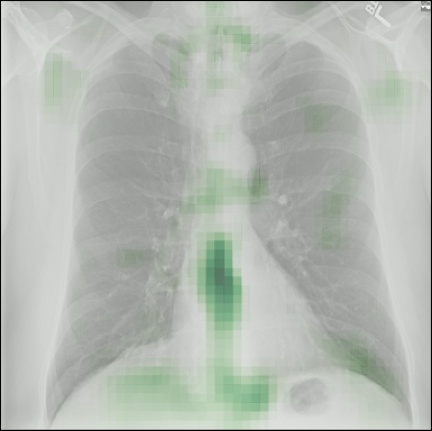

Supplement: Supplementary file 6 — Source Data [file 41467_2023_39631_MOESM6_ESM.zip › internal_maps/031220-3699_(65.0, 70.0]_69_HCC18_1_score_0.16.jpg]

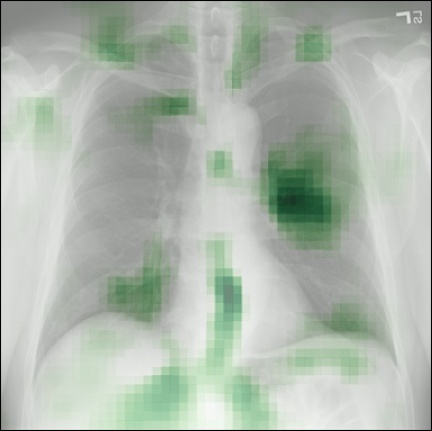

Supplement: Supplementary file 6 — Source Data [file 41467_2023_39631_MOESM6_ESM.zip › internal_maps/022311-0568_(-0.001, 58.0]_195_HCC18_0_score_0.10.jpg]

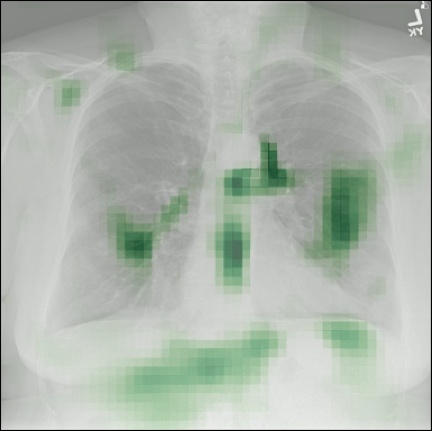

Supplement: Supplementary file 6 — Source Data [file 41467_2023_39631_MOESM6_ESM.zip › internal_maps/011616-0642_(70.0, 77.0]_84_HCC18_0_score_0.12.jpg]

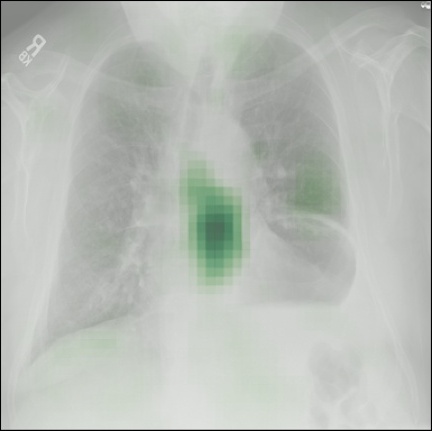

Supplement: Supplementary file 6 — Source Data [file 41467_2023_39631_MOESM6_ESM.zip › internal_maps/050319-3374_(65.0, 70.0]_155_HCC18_1_score_0.78.jpg]

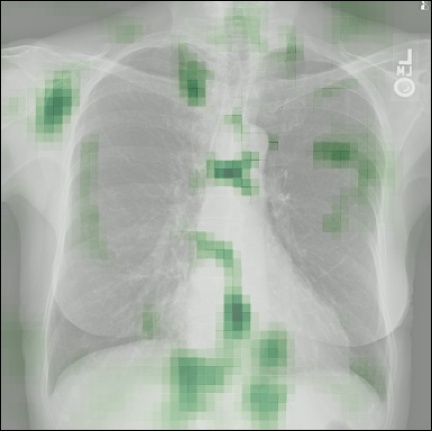

Supplement: Supplementary file 6 — Source Data [file 41467_2023_39631_MOESM6_ESM.zip › internal_maps/051421-3660_(70.0, 77.0]_36_HCC18_0_score_0.01.jpg]

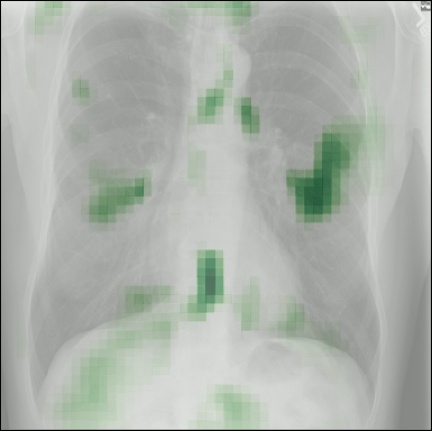

Supplement: Supplementary file 6 — Source Data [file 41467_2023_39631_MOESM6_ESM.zip › internal_maps/040720-1299_(65.0, 70.0]_133_HCC18_0_score_0.01.jpg]

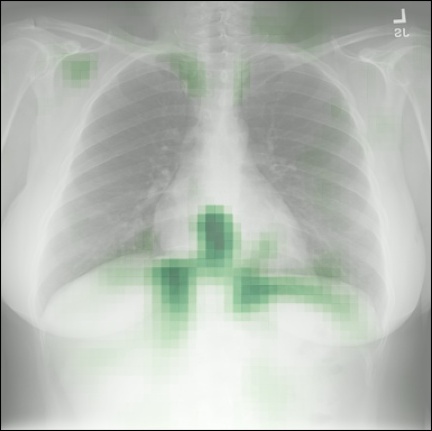

Supplement: Supplementary file 6 — Source Data [file 41467_2023_39631_MOESM6_ESM.zip › internal_maps/042913-1928_(-0.001, 58.0]_114_HCC18_1_score_0.04.jpg]

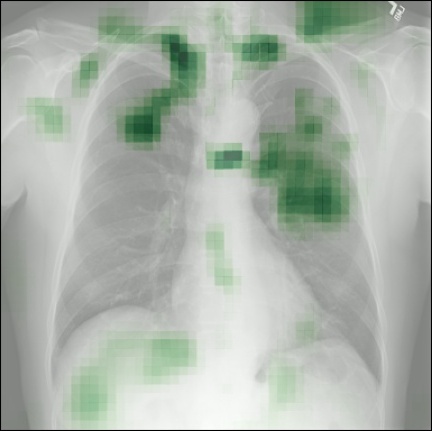

Supplement: Supplementary file 6 — Source Data [file 41467_2023_39631_MOESM6_ESM.zip › internal_maps/040611-0829_(58.0, 65.0]_192_HCC18_0_score_0.08.jpg]

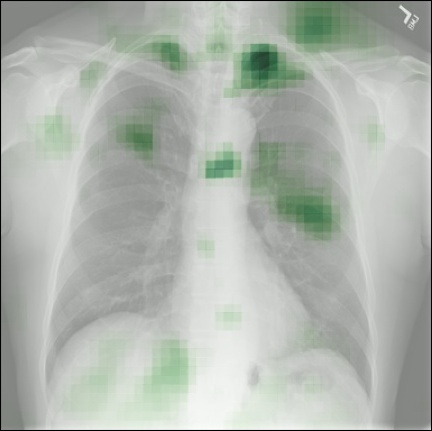

Supplement: Supplementary file 6 — Source Data [file 41467_2023_39631_MOESM6_ESM.zip › internal_maps/040211-0107_(58.0, 65.0]_188_HCC18_0_score_0.06.jpg]

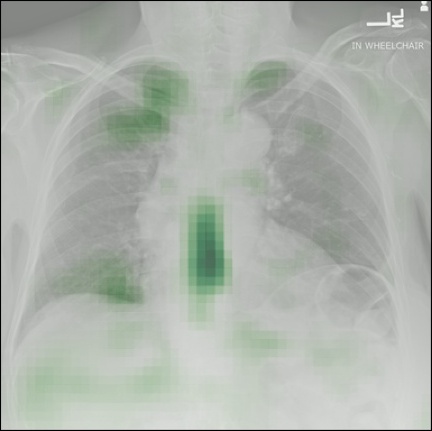

Supplement: Supplementary file 6 — Source Data [file 41467_2023_39631_MOESM6_ESM.zip › internal_maps/041321-4755_(77.0, 97.0]_22_HCC18_0_score_0.35.jpg]

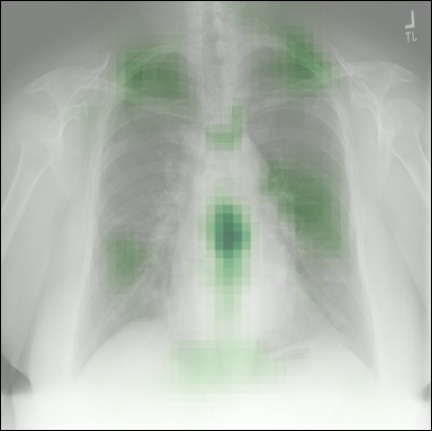

Supplement: Supplementary file 6 — Source Data [file 41467_2023_39631_MOESM6_ESM.zip › internal_maps/020713-0449_(65.0, 70.0]_175_HCC18_1_score_0.70.jpg]

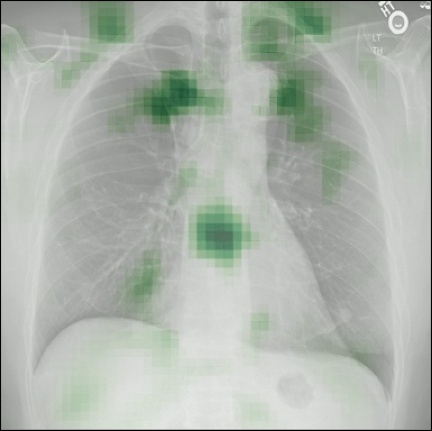

Supplement: Supplementary file 6 — Source Data [file 41467_2023_39631_MOESM6_ESM.zip › internal_maps/031115-1803_(65.0, 70.0]_109_HCC18_0_score_0.12.jpg]
